# Excessive Exogenous Gonadotropins and Genetic and Pregnancy Outcomes After Euploidy Embryo Transfer: A Secondary Analysis of a Randomized Clinical Trial

**DOI:** 10.1001/jamanetworkopen.2024.4438

**Published:** 2024-04-02

**Authors:** Tianxiang Ni, Wei Zhou, Yingbo Liu, Weiran Cui, Yang Liu, Juanjuan Lu, Qian Zhang, Zi-Jiang Chen, Yan Li, Junhao Yan

**Affiliations:** 1 Center for Reproductive Medicine, Shandong University, Jinan, Shandong, China; 2 Key Laboratory of Reproductive Endocrinology of Ministry of Education, Shandong University, Jinan, Shandong, China; 3 Shandong Key Laboratory of Reproductive Medicine, Jinan, Shandong, China; 4 Shandong Provincial Clinical Research Center for Reproductive Health, Jinan, Shandong, China; 5 Shandong Technology Innovation Center for Reproductive Health, Jinan, Shandong, China; 6 National Research Center for Assisted Reproductive Technology and Reproductive Genetics, Shandong University, Jinan, Shandong, China; 7 State Key Laboratory of Reproductive Medicine and Offspring Health, Shandong University, Jinan, Shandong, China; 8 Shanghai Key Laboratory for Assisted Reproduction and Reproductive Genetics, Shanghai, China; 9 Center for Reproductive Medicine, Ren Ji Hospital, School of Medicine, Shanghai Jiao Tong University, Shanghai, China

## Abstract

**Importance:**

The safety of exogenous gonadotropin treatment, based on its effect on embryos and pregnancy outcomes, remains inconclusive.

**Objective:**

To evaluate the associations of different doses and durations of gonadotropins with embryonic genetic status and pregnancy outcomes after euploid embryo transfer in couples with infertility.

**Design, Setting, and Participants:**

This study was a post hoc analysis of a multicenter randomized clinical trial (RCT) conducted at 14 reproductive centers throughout China from July 2017 to June 2018 that evaluated the cumulative live birth rate with or without preimplantation genetic testing for aneuploidy (PGT-A) among couples with infertility and good prognosis. The PGT-A group from the original RCT was selected for secondary analysis. Patients were divided into 4 groups according to the total dosage of exogenous gonadotropins and treatment duration: group 1 (≤1500 IU and <10 days), group 2 (≤1500 IU and ≥10 days), group 3 (>1500 IU and <10 days), and group 4 (>1 500 IU and ≥10 days). Group 1 served as the control group. Data were analyzed from June through August 2023.

**Interventions:**

Blastocyst biopsy and PGT-A.

**Main outcomes and measures:**

The primary outcomes were embryonic aneuploidy, embryonic mosaicism, and cumulative live birth rates after euploid embryo transfer.

**Results:**

A total of 603 couples (mean [SD] age of prospective mothers, 29.13 [3.61] years) who underwent PGT-A were included, and 1809 embryos were screened using next-generation sequencing. The embryo mosaicism rate was significantly higher in groups 2 (44 of 339 embryos [13.0%]; adjusted odds ratio [aOR], 1.69 [95% CI, 1.09-2.64]), 3 (27 of 186 embryos [14.5%]; aOR, 1.98 [95% CI, 1.15-3.40]), and 4 (82 of 651 embryos [12.6%]; aOR, 1.60 [95% CI, 1.07-2.38]) than in group 1 (56 of 633 embryos [8.8%]). There were no associations between gonadotropin dosage or duration and the embryo aneuploidy rate. The cumulative live birth rate was significantly lower in groups 2 (83 of 113 couples [73.5%]; aOR, 0.49 [95% CI, 0.27-0.88]), 3 (42 of 62 couples [67.7%]; aOR, 0.41 [95% CI, 0.21-0.82]), and 4 (161 of 217 couples [74.2%]; aOR, 0.53 [95% CI, 0.31-0.89]) than in group 1 (180 of 211 couples [85.3%]).

**Conclusions and relevance:**

In this study, excessive exogenous gonadotropin administration was associated with increased embryonic mosaicism and decreased cumulative live birth rate after euploid embryo transfer in couples with a good prognosis. These findings suggest that consideration should be given to minimizing exogenous gonadotropin dosage and limiting treatment duration to improve embryo outcomes and increase the live birth rate.

**Trial Registration:**

ClinicalTrials.gov Identifier: NCT03118141

## Key Points

**Question:**

Are exogenous gonadotropins associated with embryonic genetic status and pregnancy outcomes after euploid embryo transfer?

**Findings:**

In this secondary analysis of a randomized clinical trial of 603 couples with good prognosis who underwent preimplantation genetic testing for aneuploidy, an increased proportion of embryo mosaicism and a decreased cumulative live birth rate after euploid embryo transfer were observed in patients receiving high doses (>1500 IU) and prolonged treatment (≥10 days) with gonadotropins.

**Meaning:**

These results suggest that it may be beneficial to minimize exogenous gonadotropin dosage and limit treatment duration to improve embryo outcomes and the live birth rate.

## Teaser

This secondary analysis of a randomized clinical trial investigated the association of gonadotropin dosage and duration with embryonic genetic status and pregnancy outcomes among pregnant individuals who underwent euploidy embryo transfer.

## Introduction

During in vitro fertilization (IVF), gonadotropins are the main medications used for controlled ovarian hyperstimulation (COH).^1^ Continuous high-dose administration of gonadotropins can induce and maintain the growth of multiple follicles, allowing a large number of oocytes to be retrieved for IVF.^2^

However, the simultaneous development of multiple follicles and consequent high estrogen concentrations may harm oocytes, thus affecting embryonic development and pregnancy outcomes.^3,4,5,6,7^ The genetic status of the embryo is critical for embryonic development and implantation. It can be assessed using preimplantation genetic testing (PGT). High doses of exogenous gonadotropins have been reported to increase the incidence of chromosomal abnormalities, which may be the basis for impaired embryonic development after ovarian stimulation.^8^

The safety of the COH procedure for embryonic and pregnancy outcomes remains inconclusive. A multicenter randomized clinical trial (RCT)^9^ found that high-dose exogenous gonadotropin administration impaired oocyte quality and increased rates of embryonic aneuploidy and mosaicism. A higher incidence of aneuploidy has also been observed in women in the United Arab Emirates who received higher doses of exogenous gonadotropin.^10^ Nevertheless, recent studies have reported conflicting results. Wu et al^11^ reported no association between total exogenous gonadotropin dose and blastocyst aneuploidy or live birth rate in Chinese women undergoing PGT cycles. Another study^12^ found that gonadotropin dose, duration of ovarian stimulation, estradiol concentration, follicle size on the trigger day, and number of eggs retrieved were not associated with aneuploidy rate or live birth rate after euploid embryo transfer.

Notably, previous studies have included prospective mothers with a poor prognosis who had a high risk of embryonic abnormalities, such as those with advanced maternal age, recurrent miscarriages, or recurrent implantation failure. This may have a confounding effect on the accuracy of findings. Previous studies have failed to comprehensively follow up on pregnancy and neonatal complications. Furthermore, the application of next-generation sequencing (NGS) may be more effective than previously used techniques at detecting embryonic mosaicism.^13^ This study aimed to clarify the associations of different doses and treatment durations of gonadotropins with embryonic chromosomal abnormalities and pregnancy outcomes and complications in couples with young prospective mothers with infertility who had a good prognosis.

## Methods

### Study Design and Setting

This study was a post hoc analysis of results of a multicenter RCT. The original trial was conducted by Shandong University and 13 other reproductive centers in China from July 2017 to June 2018. The original study and secondary analyses were approved by the ethics committees of the Reproductive Medicine Center of Shandong University and the other reproductive centers. Signed informed consent was obtained from all couples. The Consolidated Standards of Reporting Trials (CONSORT) reporting guideline was followed. The clinical trial registration number is NCT03118141. Details of the RCT have previously been described.^14^ The trial protocol is provided in Supplement 1.

### Study Population

**Figure 1.**
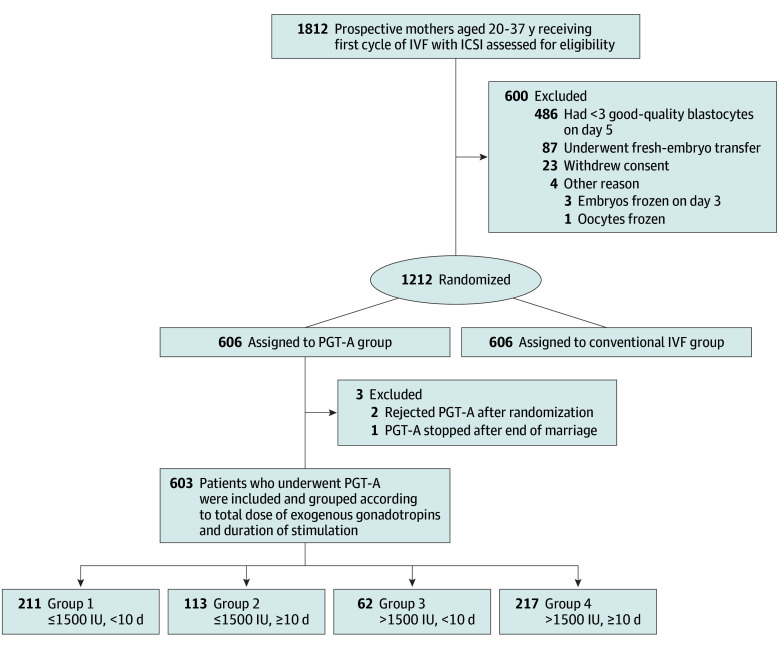
Study Flowchart ICSI indicates intracytoplasmic sperm injection; IVF, in vitro fertilization; PGT-A, preimplantation genetic testing for aneuploidy.

The study flowchart is shown in Figure 1. This trial included couples with infertility and a good prognosis, which was defined as a maternal age of 20 to 37 years and the availability of 3 or more good-quality blastocysts, with a score of 4BC or better on day 5 of embryo culture according to Gardner criteria.^15^ We selected the preimplantation genetic testing for aneuploidy (PGT-A) group from the original RCT for secondary analysis. Based on the original exclusion criteria,^14^ we excluded 3 more patients who did not undergo PGT-A, including 2 patients who rejected PGT-A after randomization and 1 patient who stopped PGT-A due to the end of their marriage after randomization.

Couples were divided into the following 4 groups according to the total dose of exogenous gonadotropins and the treatment duration: group 1 (gonadotropin dosage ≤1500 IU and treatment duration <10 days), group 2 (gonadotropin dosage ≤1500 IU and treatment duration ≥10 days), group 3 (gonadotropin dosage >1500 IU and treatment duration <10 days), and group 4 (gonadotropin dosage >1500 IU and treatment duration ≥10 days). Group 1 served as the control group.

### Procedure

COH was conducted with 3 protocols, namely a gonadotropin-releasing hormone (GnRH) agonist long protocol, a GnRH short protocol, and a GnRH antagonist protocol. The protocol was chosen mainly based on the patient’s possible ovarian response considering age, body mass index (BMI; calculated as weight in kilograms divided by height in meters squared), and ovarian reserve (eg, antral follicle count, antimüllerian hormone [AMH] concentration, and basal sex hormone concentrations). The long protocol involved the use of a GnRH agonist from the midluteal phase of the preceding menstrual cycle. Gonadotropins were given when pituitary desensitization was satisfactory until the trigger day. The short protocol involved the use of a GnRH agonist on days 2 to 3 of the menstrual period and gonadotropin administration approximately 1 to 2 days later until the trigger day. The antagonist protocol involved the addition of GnRH antagonists when the diameter of the largest follicle exceeded 12 mm. Exogenous gonadotropins used in our study included recombinant follicle-stimulating hormone (FSH) and urinary human menopausal gonadotropin. Oocyte maturation was triggered with human chorionic gonadotropin, GnRH agonist, or both when at least 2 follicles reached a mean diameter of 18 mm or greater. Oocyte retrieval was performed via transvaginal ultrasound guidance 34 to 36 hours after triggering. Fertilization was performed using intracytoplasmic sperm injection for all patients. Blastocysts were scored according to Gardner criteria.^14^

For eligible patients, 3 good-quality blastocysts were selected for each patient based on morphological scores, and these blastocysts were subjected to trophectoderm biopsy using the laser method, followed by PGT-A analysis using NGS. Specific biopsy methods and PGT-A procedures have been described in detail by Yan et al.^14^

All embryos were cryopreserved, and a single euploid embryo was chosen for each transfer. Endometrium preparation was performed with a natural ovulation cycle, an artificial regimen, or an ovulation-induction cycle. Luteal support was administered according to the standard practice at the hospital until 12 weeks of gestation. The same luteal support protocol was usually used for the corresponding endometrial preparation regimen. All pregnancies up to 3 embryo transfers within 1 year were followed up, and all pregnancy and neonatal outcomes were recorded in detail.

### Outcomes

The main outcomes were rates of embryo aneuploidy and mosaicism and cumulative live birth rate after up to 3 transfers within 1 year after randomization. Other outcomes included cumulative proportions of patients with biochemical pregnancy, clinical pregnancy, and ongoing pregnancy; cumulative risk of pregnancy loss; and incidence of maternal and neonatal complications. All outcomes in groups 2, 3, and 4 were compared with those in group 1.

A live birth was defined as the delivery of a viable infant after 28 weeks of gestation or longer. The cumulative live birth rate was calculated by dividing the number of patients who had a pregnancy of 28 weeks or more and achieved a live birth after the transfer of all euploid embryos within 1 year after randomization by the total number of patients assigned to the same group.

### Statistical Analysis

Continuous baseline data are presented as means and SDs. Differences were determined using the Mann-Whitney *U* test for data with a nonnormal distribution, and the overall *P* value was calculated using the Kruskal-Wallis test. Categorical variables are presented as frequencies and percentages, and differences were determined using the χ^2^ test. Considering that each patient had 3 embryos tested using PGT-A, we performed generalized estimating equation analysis to evaluate the effect of the total dosage and duration of gonadotropin treatment on embryonic genetic status. Confounding factors included maternal age, BMI, AMH concentration, ovarian stimulation protocol, and trigger type. Binary logistic regression analysis was used to investigate the relationship between the total dosage and duration of gonadotropin treatment and the cumulative live birth rate. Corresponding confounding factors included maternal age, BMI, AMH concentration, and endometrial thickness on the trigger day. A 2-sided *P* value < .05 was considered to indicate statistical significance. Statistical analyses were performed using SPSS statistical software version 26.0 (IBM). Data were analyzed from June through August 2023.

## Results

### Baseline Characteristics, COH Outcomes, and Embryo Culture

A total of 603 couples (mean [SD] age of prospective mothers, 29.13 [3.61] years) with a good prognosis who underwent PGT-A were included in the analysis, and 1809 embryos were screened using NGS. There were 211 couples with 633 embryos tested in group 1, 113 couples with 339 embryos tested in group 2, 62 couples with 186 embryos tested in group 3, and 217 couples with 651 embryos tested in group 4. As shown in Table 1, basic characteristics of patients in groups 2, 3, and 4 were comparable with those of patients in group 1. Patients in group 3 were older (mean [SD] age, 29.87 [3.91] years vs 28.55 [3.47] years; *P* = .02), had a higher mean (SD) BMI (24.69 [3.80] vs 22.18 [3.08]; *P* < .001), and had a lower mean (SD) AMH concentration (6.76 [4.25] ng/mL vs 8.12 [5.21] ng/mL; *P* = .04) than those in group 1. Patients in group 4 were also older (mean [SD] age, 29.62 [3.71] years vs 28.55 [3.47] years; *P* = .004). had a higher mean (SD) BMI (23.80 [3.49] vs 22.18 [3.08]; *P* < .001), and had a relatively poorer ovarian reserve, with a lower AMH concentration, a higher FSH concentration, and a lower antral follicle count on both sides of the ovaries than those in group 1.

**Table 1.** Baseline Characteristics of Patients, Outcomes of COH, and Embryo Culture

Characteristic	Patient measure, mean (SD) (N = 603)				Overall *P* value	*P* value vs group 1^a^		
	Group 1 (n = 211)^a^	Group 2 (n = 113)^a^	Group 3 (n = 62)^a^	Group 4 (n = 217)^a^		Group 2^a^	Group 3^a^	Group 4^a^
Age, y	28.55 (3.47)	28.85 (3.30)	29.87 (3.91)	29.62 (3.71)	.01	.50	.02	.004
BMI	22.18 (3.08)	22.04 (2.65)	24.69 (3.80)	23.80 (3.49)	<.001	.95	<.001	<.001
Ultrasonographic finding								
AFC in right ovary	11.40 (5.04)	12.13 (6.74)	11.56 (5.66)	10.57 (5.35)	.09	.99	.84	.023
AFC in left ovary	11.11 (5.28)	11.17 (6.16)	11.34 (6.23)	10.25 (5.03)	.14	.37	.92	.023
Laboratory testing								
FSH, mIU/mL	5.74 (1.42)	5.95 (1.49)	6.05 (1.54)	6.24 (1.75)	.009	.14	.23	.001
LH, mIU/mL	7.25 (5.23)	6.88 (4.92)	6.38 (4.23	6.65 (4.55	.52	.46	.16	.32
Estradiol, pg/mL	40.97 (24.96)	41.22 (25.16)	39.18 (19.43)	41.05 (29.00)	.68	.98	.49	.29
Progesterone, ng/mL	0.67 (1.58)	0.54 (0.70)	0.59 (0.89)	0.71 (1.99)	.98	.93	.73	.77
Total testosterone, ng/dL	3753 (2615)	3631 (2025)	3777 (1751)	3533 (1624)	.67	.76	.32	.10
Prolactin, ng/mL	18.18 (9.34)	18.68 (8.89)	17.48 (7.95)	16.61 (8.63)	.06	.29	.93	.12
AMH, ng/mL	8.12 (5.21)	7.56 (4.49)	6.76 (4.25)	6.33 (3.74)	.001	.36	.04	<.001
Ovarian stimulation protocol								
Long GnRH agonist protocol	72 (34.1)	68 (60.2)	14 (22.6)	132 (60.8)	<.001	<.001	.12	<.001
Short GnRH agonist protocol	43 (20.4)	9 (8.0)	11 (17.7)	15 (6.9)				
Antagonist protocol	96 (45.5)	36 (31.9)	37 (59.7)	70 (32.3)				
Starting Gn dose, IU	144.67 (28.58)	130.11 (21.88)	210.48 (38.02)	171.29 (50.23)	<.001	<.001	<.001	<.001
Gn total dosage, IU	1137.10 (204.93)	1293.05 (188.09)	1764.24 (175.75)	2294.83 (745.93)	<.001	<.001	<.001	<.001
Duration of stimulation, days	8.24 (0.81)	10.32 (0.60)	8.44 (0.59)	11.58 (1.48)	<.001	<.001	.18	<.001
Trigger type								
hCG only	138 (65.4)	90 (79.6)	33 (53.2)	181 (83.4)	<.001	.03	.03	<.001
GnRH agonist only	17 (8.1)	5 (4.4)	2 (3.2)	12 (5.5)				
GnRH agonist + hCG	56(26.5)	18(15.9)	27(43.5)	24(11.1)				
Estradiol level on hCG trigger day, pg/mL	6316.07 (2929.60)	5954.19 (2296.78)	5391.30 (2398.42)	5697.13 (2376.04)	.08	.52	.026	.07
Endometrial thickness on hCG trigger day, mm	1.03 (0.21)	1.09 (0.22)	0.98 (0.25)	1.10 (0.22)	<.001	.02	.11	<.001
No. of oocytes retrieved	20.12 (7.86)	20.00 (6.76)	21.61 (10.31)	20.01 (7.30)	.85	.79	.41	.78
No. of good-quality embryos on day 3	9.29 (4.05)	9.86 (4.70)	10.05 (5.39)	9.63 (4.50)	.89	.62	.55	.52
No. of good-quality embryos on day 5 and 6, No.	7.22 (2.93)	7.38 (3.20)	8.27 (3.94)	7.14 (2.85)	.24	.91	.06	.89
Mean No. of transfer cycles within 1 y	1.16 (0.48)	1.18 (0.49)	1.16 (0.52)	1.14 (0.51)	.85	.90	.96	.46
Mean No. of transferred embryos within 1 y	1.18 (0.50)	1.18 (0.49)	1.16 (0.52)	1.15 (0.52)	.80	.95	.91	.35

Abbreviations: AFC, antral follicle count; AMH, antimüllerian hormone; BMI, body mass index (calculated as weight in kilograms divided by height in meters squared); COH, controlled ovarian hyperstimulation; FSH, follicle-stimulating hormone; Gn, gonadotropin; GnRH, gonadotropin-releasing hormone; hCG, human chorionic gonadotropin; LH, luteinizing hormone.

SI conversion factors: To convert estradiol to picomoles per liter, multiply by 3.671; FSH and LH to international units per liter, multiply by 1.0; progesterone to nanomoles per liter, multiply by 3.18; prolactin to micrograms per liter, multiply by 1; testosterone to nanomoles per liter, multiply by 0.0347.

^a^ Group 1 had a gonadotropin dosage of 1500 IU or less and a treatment duration of less than 10 days; group 2 had a gonadotropin dosage of 1500 IU or less and a treatment duration of 10 days or more; group 3 had a gonadotropin dosage greater than 1500 IU and a treatment duration of less than 10 days; group 4 had a gonadotropin dosage greater than 1500 IU and a treatment duration of 10 days or more.

Outcomes of COH and embryo culture are presented in Table 1. As expected, stimulation protocols, starting gonadotrophin dose, and trigger type for oocyte maturation were different between groups. There was a significant increase in mean (SD) endometrial thickness on the trigger day compared with group 1 (1.03 [0.21] mm) in group 2 (1.09 [0.22] mm; *P* = .02) and group 4 (1.10 [0.22] mm; *P* < .001). In addition, endometrial preparation protocols for frozen embryo transfer were similar between groups (eTable 1 in Supplement 2).

### Association of Exogenous Gonadotrophin Treatment With Embryonic Chromosomal Abnormalities

**Figure 2.**
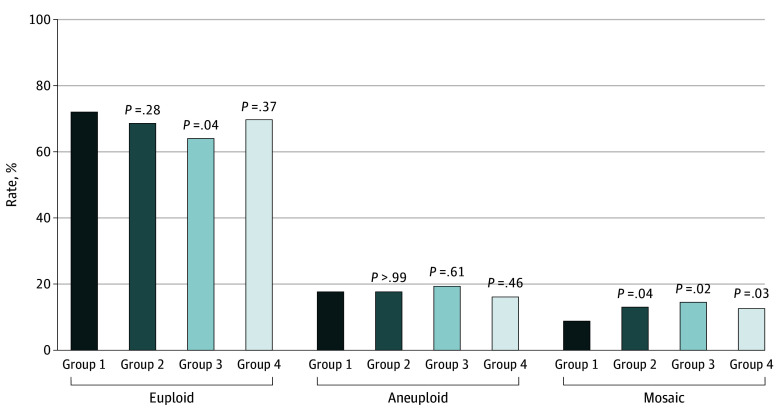
Results of Embryo Sequencing Differences in mosaic rate, aneuploidy rate, and euploidy rate are shown in this figure. Group 1 had a gonadotropin dosage of 1500 IU or less and a treatment duration of less than 10 days; group 2 had a gonadotropin dosage of 1500 IU or less and a treatment duration of 10 days or more; group 3 had a gonadotropin dosage greater than 1500 IU and a treatment duration of less than 10 days; group 4 had a gonadotropin dosage greater than 1500 IU and a treatment duration of 10 days or more. Analyses were performed using χ^2^ tests. All comparisons are vs group 1.

As presented in Figure 2 and Table 2, the percentage of mosaic embryos was significantly higher in groups 2 (44 embryos [13.0%]; adjusted odds ratio [aOR], 1.69 [95% CI, 1.09-2.64]), group 3 (27 embryos [14.5%]; aOR. 1. 98 [95% CI, 1.15-3.40]) and group 4 (82 embryos [12.6%]; aOR, 1.60 [95% CI, 1.07-2.38]) than in group 1 (56 embryos [8.8%]). In multiplicative interaction analysis, increased gonadotropin dosage was associated with an increase in the incidence of mosaicism when a short treatment duration (<10 days) was used (OR, 1.848 [95% CI, 1.12-3.04]) but not when a long treatment duration (≥10 days) was used (OR, 0.96 [95% CI, 0.64-1.42]; *P* for interaction = .04) (eTable 2 in Supplement 2). Additionally, gonadotropin dose or treatment duration did not affect the rate of embryonic aneuploidy (Figure 2; Table 2).

**Table 2.** Association of Gonadotropins Dosage and Duration of Stimulation With Embryonic Genetic Status

Genetic outcome	Embryos, No. (%) (N = 1809)	OR (95%CI)^a^	
		Crude	Adjusted^b^
Aneuploidy			
Group 1 (n = 633)^c^	112 (17.7)	1 [Reference]	1 [Reference]
Group 2 (n = 339)^c^	60 (17.7)	1.05 (0.73-1.52)	1.01 (0.69-1.47)
Group 3 (n = 186)^c^	36 (19.4)	1.23 (0.76-1.99)	1.25 (0.78-2.01)
Group 4 (n = 651)^c^	105 (16.1)	0.94 (0.70-1.27)	0.91 (0.66-1.25)
Mosaicism			
Group 1 (n = 633)^c^	56 (8.8)	1 [Reference]	1 [Reference]
Group 2 (n = 339)^c^	44 (13.0)	1.54 (1.01-2.35)	1.69 (1.09-2.64)
Group 3 (n = 186)^c^	27 (14.5)	1.85 (1.121-3.04)	1.98 (1.15-3.40)
Group 4 (n = 651)^c^	82 (12.6)	1.47 (1.02-2.35)	1.60 (1.07-2.38)

Abbreviation: OR, odds ratio.

^a^ Associations investigated by generalized estimating equations.

^b^ Adjusted for age, body mass index (calculated as weight in kilograms divided by height in meters squared), antral follicle count, ovarian stimulation protocol, and trigger type.

^c^ Group 1 had a gonadotropin dosage of 1500 IU or less and a treatment duration of less than 10 days; group 2 had a gonadotropin dosage of 1500 IU or less and a treatment duration of 10 days or more; group 3 had a gonadotropin dosage greater than 1500 IU and a treatment duration of less than 10 days; group 4 had a gonadotropin dosage greater than 1500 IU and a treatment duration of 10 days or more.

### Association of Exogenous Gonadotrophin Treatment With Pregnancy Outcomes After PGT-A

Pregnancy outcomes are shown in Table 3 and eTable 3 in Supplement 2. The cumulative live birth rate was significantly lower in group 2 (83 couples [73.5%]; aOR, 0.49 [95% CI, 0.27-0.88]), group 3 (42 couples [67.7%]; aOR, 0.41 [95% CI, 0.21-0.82]), and group 4 (161 couples [74.2%]; aOR, 0.53 [95% CI, 0.31-0.89]) than in group 1 (180 couples [85.3%]). There was also a significant decrease in the cumulative proportion of patients with ongoing pregnancy in groups 2, 3, and 4 compared with group 1 (Table 3). In group 3 compared with group 1, the cumulative proportion of patients with biochemical pregnancy (49 individuals [79.0%] vs 192 individuals [91.0%]; aOR, 0.37 [95% CI, 0.16-0.82]) and the cumulative proportion of patients with clinical pregnancy (44 individuals [71.0%] vs 189 individuals [89.6%]; aOR, 0.29 [95% CI, 0.14-0.60]) were decreased and the incidence of pregnancy loss rate among individuals with biochemical pregnancy was increased (8 individuals [16.3%] vs 6 individuals [3.1%]; aOR, 5.88 [95% CI, 1.82-19.02]).

**Table 3.** Odds of Pregnancy Outcomes

Outcome	Patients, No. (%) (N = 603)				aOR vs group 1 (95% CI)^a^		
	Group 1 (n = 211)^b^	Group 2(n = 113)^b^	Group 3 (n = 62)^b^	Group 4 (n = 217)^b^	Group 2	Group 3	Group 4
Primary outcome: cumulative live birth rate^c^	180 (85.3)	83 (73.5)	42 (67.7)	161 (74.2)	0.49 (0.27-0.88)	0.41 (0.21-0.82)	0.53 (0.31-0.89)
Secondary outcomes^c^							
Cumulative biochemical pregnancy	192 (91.0)	95 (84.1)	49 (79.0)	188 (86.6)	0.55 (0.27-1.12)	0.37 (0.16-0.82)	0.67 (0.35-1.29)
Cumulative clinical pregnancy	189 (89.6)	92 (81.4)	44 (71.0)	178 (82.0)	0.52 (0.27-1.01)	0.29 (0.14-0.60)	0.54 (0.30-0.97)
Cumulative ongoing pregnancy	184 (87.2)	86 (76.1)	42 (67.7)	165 (76.0)	0.48 (0.26-0.88)	0.35 (0.18-0.71)	0.51 (0.29-0.87)
Cumulative pregnancy loss^d^							
Biochemical	6 (3.1)	6 (6.3)	8 (16.3)	11 (5.9)	2.22 (0.68-7.19)	5.88 (1.82-19.02)	2.12 (0.74-6.90)
Clinical	14 (7.3)	11 (11.6)	2 (4.1)	19 (10.1)	1.66 (0.69-4.00)	0.21 (0.03-1.74)	1.24 (0.57-2.74)

Abbreviation: aOR, adjusted odds ratio.

^a^ The analysis was conducted using binary logistic regression. ORs were adjusted for age, body mass index (calculated as weight in kilograms divided by height in meters squared), antral follicle count, and endometrial thickness on trigger day. All comparisons were calculated with group 1 as the reference.

^b^ Group 1 had a gonadotropin dosage of 1500 IU or less and a treatment duration of less than 10 days; group 2 had a gonadotropin dosage of 1500 IU or less and a treatment duration of 10 days or more; group 3 had a gonadotropin dosage greater than 1500 IU and a treatment duration of less than 10 days; group 4 had a gonadotropin dosage greater than 1500 IU and a treatment duration of 10 days or more.

^c^ Percentages are out of the total number of patients in this subgroup.

^d^ Percentages are out of the number of cumulative biochemical pregnancies in this subgroup.

### Association of Exogenous Gonadotrophin Treatment With Maternal and Neonatal Complications After PGT-A

Maternal and neonatal outcomes after PGT-A are shown in eTable 4 in Supplement 2. Frequencies of maternal complications in groups 2, 3, and 4 were similar to those in group 1. There were significantly more babies with a low birth weight in group 2 than in group 1 (8 of 83 babies with birth weight <2500 g [9.6%] vs 4 of 180 babies with birth weight < 2500 g [2.2%], OR, 4.69 [95% CI, 1.37-16.06]).

## Discussion

In this secondary analysis of an RCT, we investigated the association of high-dose administration of exogenous gonadotropins with embryonic and pregnancy outcomes after euploidy embryo transfer in patients with a good prognosis. We found that patients who received higher gonadotropin doses and a longer treatment duration had a higher proportion of mosaic embryos and a lower cumulative live birth rate.

Previous studies on the effect of exogenous gonadotropin treatment have yielded contradictory results. Initially, it was found that high-dose exogenous gonadotropin treatment was associated with increased oocyte meiosis errors and thus more aneuploid oocytes.^16,17^ Rubio et al^8^ discovered that the ovarian stimulation regimen had dose-dependent adverse effects on oocyte quality and fetal aneuploidy, whereas reducing the dosage of gonadotropins in individuals with high response increased the fertilization rate and embryo quality. Considering that fluorescence in situ hybridization was used to evaluate fetal aneuploidy in these 3 studies, only several chromosomes were analyzed and the genetic status of the embryos could not be fully evaluated. However, some recent studies using comprehensive chromosome sequencing techniques for PGT, such as array comparative genomic hybridization, found that exogenous gonadotropin dosage was not associated with adverse embryo aneuploidy outcomes. Wu et al,^11^ Irani et al,^12^ and Barash et al^18^ found that high gonadotropin doses were not associated with aneuploidy rate or pregnancy outcomes after a single embryo transfer for each PGT cycle. Sekhon et al^19^ investigated the association of different doses of gonadotropin with fetal aneuploidy in 2017 and found that in patients with normal ovarian responsiveness (ovulation induction time <12 days), the use of gonadotropins was not associated with a change in the embryonic aneuploidy rate. However, for patients with decreased ovarian reactivity (ovulation promotion time ≥12 days), the incidence of embryo aneuploidy increased with the increase in gonadotropin dosage. Consistent with these findings, we also found that neither the gonadotropin dose nor the treatment duration increased the risk of embryonic aneuploidy.

With the development of NGS techniques, which have delivered important improvements in diagnostic accuracy and sensitivity for PGT-A, it is now possible to detect more mosaic embryos than previously detected using array comparative genomic hybridization.^13^ We found that in patients undergoing IVF who had a good prognosis, high doses of gonadotrophins or prolonged treatment significantly increased the risk of embryonic mosaicism. Notably, the effect of gonadotropin dosage on the incidence of mosaicism was significant with a short treatment duration but not a long duration. The higher proportion of mosaic embryos may be due to the impaired potential for embryonic development caused by high concentrations of gonadotropins or the inability of aneuploid cells to be eliminated by self-correction mechanisms. However, Coll et al^20^ used a logistic mixed multivariable model to study patients with a poor prognosis, including those with advanced maternal age, recurrent miscarriage, recurrent implantation failure, and male factors, and found that the total gonadotropin dose was not associated with the incidence of mosaic embryos. Of note, the previously mentioned conditions are the main contributors to embryo abnormalities and may have obfuscated the effects of other factors. Furthermore, subgroup analyses according to dose and duration of gonadotropin treatment were not performed in the previously mentioned studies.

Although Wu et al^11^ and Barash et al^18^ found no association of high-dose exogenous gonadotropin treatment with pregnancy outcomes after PGT-A, different conclusions have been reported in other studies. Several studies have found that exogenous gonadotropin treatment was associated with adverse outcomes in embryo implantation and postimplantation development in hamsters and rats.^3,4,5,6,7^ A study involving 658 519 fresh autologous IVF cycles in patients with a good prognosis by Baker et al^1^ found that the live birth rate decreased significantly with increasing FSH dose, independent of the number of oocytes obtained. In 2 small retrospective studies,^21,22^ high doses of gonadotropin were associated with lower rates of clinical pregnancy and live births, along with a higher miscarriage rate. These associations were independent of age, basal FSH concentration, endometrial thickness, maximum estradiol concentrations, number of eggs retrieved, and type of embryo transfer. These findings are consistent with the poor pregnancy outcomes observed in the high-dose and long-term treatment group in our study, in which the proportion of patients achieving ongoing pregnancies and live birth were significantly lower than corresponding values in the low-dose and short-term stimulation group.

In addition, a Dutch population-based study^23^ found that birth weights of siblings conceived by IVF were not significantly different from those of their naturally conceived siblings and that low maternal fertility was associated with increased risk of having an infant with low birth weight. The longer treatment duration and increased dosage in this study represent increasing infertility of the patient, which may explain the increased proportion of babies with low birth weight in group 2 in our study.

### Strengths and Limitations

Patients included in this study were highly homogeneous and were young, with a good prognosis for live birth. Data for this population have not previously been reported. We separately analyzed effects of exogenous gonadotropin treatment on the incidence of aneuploidy and mosaicism based on NGS analysis. We also report detailed information regarding pregnancy and newborn complications to comprehensively reflect the safety of exogenous gonadotropin administration. However, this study also has several limitations. First, this was a secondary analysis, and the original RCT was not specifically designed to evaluate effects of gonadotropins on embryonic genetic status and live birth rate. Large, appropriately designed cohort studies are warranted to verify our conclusions. Second, only 3 embryos were tested for each patient, and at most only 3 transfer cycles were analyzed for cumulative live birth rate. Third, false-positive and false-negative results of trophectoderm biopsies and NGA analysis are unavoidable, such as potential inconsistencies between sequencing results of the trophectoderm and the inner cell mass, especially when detecting mosaicism.^24^ Fourth, there were some differences between groups, such as different stimulation protocols (including GnRH agonist vs antagonist) and different trigger types, which may have conferred confounding effects. However, we adjusted for these confounding factors using regression analyses. Other factors, such as the type of gonadotropin used, were not included in this analysis due to a lack of information.

## Conclusions

In this secondary analysis of the results of a multicenter RCT, excessive exogenous gonadotropins were associated with increase incidence of embryonic mosaicism and decrease the cumulative live birth rate after euploid embryo transfer in couples with a good prognosis. These findings suggest that consideration should be given to minimizing exogenous gonadotropin dosage and limiting treatment duration to improve embryonic outcomes and increase the live birth rate.

## Notes

Supplement 1.
Trial Protocol and Statistical Analysis Plan

Supplement 2. **eTable 1.** Endometrial Preparation Protocols for Frozen Embryo Transfers **eTable 2.** Interaction Between Total Dosage and Duration of Gonadotropins and the Effect on Embryo Mosaicism **eTable 3.** Binary Logistic Regression of the Effect of Gonadotropin Dosage and Treatment Duration on Cumulative Live Birth Rate **eTable 4.** Maternal and Neonatal Complications

Supplement 3.
Data Sharing Statement
