# Supplementary material for: Excessive Exogenous Gonadotropins and Genetic and Pregnancy Outcomes After Euploidy Embryo Transfer: A Secondary Analysis of a Randomized Clinical Trial
Source: JAMA Netw Open. 2024 Apr 2;7(4):e244438. doi: 10.1001/jamanetworkopen.2024.4438 (PMC10988349; doi:10.1001/jamanetworkopen.2024.4438)
Supplement: Supplement 1. — Trial Protocol and Statistical Analysis Plan [file jamanetwopen-e244438-s001.pdf]

Protocol

This trial protocol has been provided by the authors to give readers additional information about the work.

Protocol for: Yan J, Qin Y, Zhao H, et al. Live birth with or without preimplantation genetic testing for aneuploidy.
N Engl J Med 2021;385:2047-58. DOI: 10.1056/NEJMoa2103613

There is the only one version of the protocol, and the implementation process of this trial was strictly in accordance with it.

The statistical analysis plan was finalized before starting enrollment, and was described in section STATISTICAL CONSIDERATIONS (Page 39-40) of study protocol, and as follows:

The primary analysis will be performed in accordance with the principle of intent-to-treat. Continuous variables will be described as mean ± standard deviation for the normally distributed variables and as median and range for the non-normally distributed variables. Category variables will be presented as frequency and percentage. The differences in the cumulative live birth rate in the two treatment arms will compared by absolute risk difference and its 95% confidence interval (CI). The relative risk and its 95% CI will also be calculated. Non-superiority of PGS to IVF will be considered to be established if the upper limit of the 95% CI of absolute risk difference was shown to lie below the non-superiority margin of 7%.

The differences in the secondary outcomes such as rate of good birth outcome, cumulative pregnancy rate, cumulative pregnancy loss rate, multiple pregnancy rate obstetric and neonatal complications will be tested by Person chi-square analyses. The absolute difference and 95%CI as well as relative risk and 95% CI will also be calculated. The difference between duration of pregnancy, birth weight and times of embryo transfer will be tested by student t test and non-parameter test if necessary.

The tertiary outcomes such as the rates of pregnancy, pregnancy loss and live birth after the first transfer will be analyzed with Pearson chi-square test. Cost-effectiveness analysis will be done by decision analytic model with TreeAge Pro 2014 (2014 Version; TreeAge software). Cost effectiveness is defined as an incremental cost effectiveness ratio (ICER).The ICER for live birth was calculated as the difference in cost between two treatment groups divided by the difference in live birth rates between the two groups. The secondary analysis will be performed according to per-protocol analyses. The primary and secondary outcomes will be compared according to actual treatment that subjects received and among subjects who complete embryo transfer(s).

Subgroup analyses will be performed according to different stimulation protocol (e.g. agonist long, agonist short, and antagonist protocol), different endometrial preparation protocol for FET (e.g. natural ovulation cycle and artificial cycle), age groups (e.g. 20~30 group, 30~35 group and >35 group), with or without history of recurrent spontaneous abortion.

Any deviations from the previously described statistical plan will be described and justified in a protocol amendment.

Cumulative live birth rate with eSET after in-vitro fertilization with preimplantation genetic screening by next-generation sequencing versus conventional in-vitro fertilization: A pragmatic randomized controlled clinical trial (CESE-PGS)

Study Chair:

Zi-Jiang Chen, M.D., Ph.D.
Vice President, Shandong University
Chief, Reproductive Medical Center, Shandong University

Study Intervention Provided by:

N.A.

Sponsor of IND (IDE):

N.A.

Version 1.0
March 07, 2017

Protocol Committee:

Name	Affiliation	Email
Zi-Jiang Chen	Center for Reproductive Medicine, Shandong University	chenzijiang@vip.163.com
Richard S Legro	Department of Obstetrics and Gynecology, Penn State College of Medicine	Rsl1@psu.edu
Heping Zhang	Department of Biostatistics, Yale University School of Public Health	heping.zhang@yale.edu
Jiayin Liu	Department of Obstetrics and Gynecology, First Affiliated Hospital of Nanjing Medical University	jyliu_nj@126.com
Junhao Yan	Center for Reproductive Medicine, Shandong University	yyy306@126.com
Xiaoxi Sun	ShangHai JiAi Genetics & IVF Institute	
Yimin Zhu	Department of Reproductive Endocrinology, Women's Hospital, School of Medicine, Zhejiang University	zhuyim@zju.edu.cn
Yun Sun	Center for Reproductive Medicine, Ren Ji Hospital, School of Medicine, Shanghai Jiao Tong University	syun163@163.com
Yuhua Shi	Center for Reproductive Medicine, Shandong University	shiyuhua2003@126.com
Yuan Gao	Center for Reproductive Medicine, Shandong University	gaoyuan@sduivf.com
Keliang Wu	Center for Reproductive Medicine, Shandong University	wukeliang_527@163.com
Daimin Wei	Center for Reproductive Medicine, Shandong University	sdweidaimin@163.com

Steering Committee:

Name
Zi-Jiang Chen
Richard S Legro
Heping Zhang
Yun Sun
Junhao Yan
Yuhua Shi
Daimin Wei

Data Safety Monitoring Board (DSMB):

Name	Specialty	Affiliation	Role in DSMB
Joe Leigh Simpson	Genetics	Preimplantation Genetic Diagnosis International Society (PGDIS), Chicago, IL, USA	Chair of DSMB
Robert Rebar	Reproductive Medicine, Ethics	Department of Obstetrics and Gynecology, Western Michigan University Homer Stryker	Voting member
TC Li	Reproductive Medicine	Reproductive Medicine and Surgery Unit, Prince of Wales Hospital, Chinese University of Hong Kong	Voting member
Jun Zhang	Statistics	Shanghai Xinhua Hospital	Voting member
Cong Zhang	Defender of subjects' interests	Shandong Normal University	Voting member

Table of Abbreviations and definitions in alphabetical order

Abbreviations	Definitions
ACOG	American Congress of Obstetrician and Gynecologists
aCGH	array-comparative genome hybridization
AMA	advanced maternal age
ART	assisted reproductive technology
ASRM	American Society for Reproductive Medicine
BAFC	bilateral antral follicle count
BFM	BlueFuse Multi
BFS	British Fertility Society
BMI	body mass index
CCS	comprehensive chromosome screening
CDC	Centers for Disease Control and Prevention
CI	confidence interval
COH	controlled ovarian hyperstimulation
CRF	case report form
DCC	Data Coordinator Center
DET	double embryo transfer
DNA	deoxyribonucleic acid
DSMB	Data and Safety Monitoring Board
eSET	elective single embryo transfer
E2	Estradiol
ESHRE	European Society for Human Reproduction and Embryology
FET	frozen embryo transfer
FISH	fluorescence in situ hybridization
FSH	follicle stimulating hormone
GnRH	gonadotropin releasing hormone
HCG	human chorionic gonadotropin
HMG	human menopausal gonadotropin
HSG	Hysterosalpingography
ICM	inner cell mass
ICSI	intracytoplasmic sperm injection
IR	implantation rates
IRB	institutional Review Board
IVF	in-vitro fertilization
LBR	live birth rate
LH	luteinizing hormone
MDA	multiple displacement amplification
MESA	microsurgical epididymal sperm aspiration
NGS	next generation sequencing
OHSS	ovarian hyperstimulation syndrome
OS	observational studies
P	Progesterone

PCOS	polycystic ovary syndrome
PGD	preimplantation genetic diagnosis
PGS	preimplantation genetic screening
PRL	Prolactin
q-PCR	quantitative polymerase chain reaction
RCT	randomized controlled trial
RIF	recurrent implantation failure
RM	recurrent miscarriage
SAE	serious adverse event
SID	study identification number
SNP	single nucleotide polymorphism
T	Testosterone
TE	Trophectoderm
TESE	testicular sperm extraction
TSH	thyroid stimulating hormone
WGA	whole genome amplification
ZP	zona pellucida

Phrases

Good Birth Outcome defined as a live birth of an infant born at ≥ 37 weeks, with a birth weight between 2500 and 4000g and without a major congenital anomaly

TABLE OF CONTENTS

Page

SYNOPSIS ……….…………………………………………………………………... 8

1. STUDY OBJECTIVES ………………………………………………………... 11

1.1 Primary Objective ……………………………………………….………….. 11
1.2 Secondary Objectives...………………………………………………..………..11

2. BACKGROUND ……………………………………………………………..….. 11

2.1 Rationale ………...…………………………………………………………..… 11
2.2 Supporting Data …… ………………………………..……………………….12

3. STUDY DESIGN ……………………………………………………..…………..19

4. SELECTION AND ENROLLMENT OF SUBJECTS …………………...…………..19

4.1 Inclusion Criteria ……………………………………………………..………..19
4.2 Exclusion Criteria ………………………………………………………..…….19
4.3 Study Enrollment Procedures …………………………………………….………20

5. STUDY INTERVENTIONS …………………………………………….………..20

5.1 Interventions, Administration, and Duration ..………………………….………..20
5.2 Handling of Study Interventions ……………………………………….………24
5.3 Concomitant Interventions ………………………………………………..…….25
5.4 Adherence Assessment …………………………………………………………....27

6. CLINICAL AND LABORATORY EVALUATIONS ………………………………..27

6.1 Schedule of Evaluations ……………………………………………….…………28
6.2 Timing of Evaluations ……………………………………………………….……29
6.3 Special Instructions and Definitions of Evaluations …………………….………..31

7. MANAGEMENT OF ADVERSE EXPERIENCES ……...…………………...…….33

8. CRITERIA FOR INTERVENTION DISCONTINUATION ……………...……….35

9. STATISTICAL CONSIDERATIONS ……………………………………...……..35

9.1 General Design Issues …………………………………………………..………...35
9.2 Outcomes …………………………………………………………………...……….36

9.3 Sample Size and Accrual ……………………………………………………..………..37
9.4 Data Monitoring ………………………………………………………………..……...37
9.5 Data Analyses …………………………………………………………...……..38

10. DATA COLLECTION, SITE MONITORING, AND ADVERSE EXPERIENCE REPORTING …………………………………………………………………...39

10.1 Records to be Kept …………………………………………………………..……39
10.2 Role of Data Management …………………………………………..……39
10.3 Quality Assurance ……………………………………………………..…40
10.4 Adverse Experience Reporting ……………………………………..…40

11. HUMAN SUBJECTS ………………………………………………………..……41

11.1 Institutional Review Board (IRB) Review and Informed Consent ………………..….41
11.2 Subject Confidentiality ………………………………………………………..……..41
11.3 Study Modification/Discontinuation …………………………….…..……42

12. PUBLICATION OF RESEARCH FINDINGS ……………………………...……..42

13. REFERENCES ………………………………………………………...………..42

14. APPENDICES…………………………………………………………………....46

I. Table 1. Comparison among different methods for PGS……………………………46
II. Table 3. List of registered randomized clinical trial regrading PGS……………………49
III. Informed consent……………………………………………………………………..91
IV. Questionnaire (Ferti QoL)…………………………………………………………98

SYNOPSIS

Study Title

Cumulative live birth rate with eSET after in-vitro fertilization with preim-plantation genetic screening by next-generation sequencing versus conven-tional in-vitro fertilization: A pragmatic randomized controlled clinical trial (CESE-PGS)

Rationale

PGS is widely being utilized to increase the chance of pregnancy in women with a good prognosis for pregnancy. However, there is still insufficient evidence of the risk/benefit ratio of PGS compared with conventional IVF. Existing trials are underpowered and have not completely tracked the outcomes of public health interest, i.e. live birth, anomalies, and maternal/fetal/infant complications.

Objectives

The primary objective is to compare the cumulative live birth rate of in-vitro fertilization (IVF) with pre-implantation genetic screening (PGS group) versus IVF alone (IVF group) in good-prognosis women with indications for IVF.

The secondary objectives are to compare the rate of a Good Birth Outcome (defined as a live birth of an infant born at ≥ 37 weeks, with a birth weight between 2500 and 4000g and without a major congenital anomaly), cumulative pregnancy rate, cumulative pregnancy loss rate, multiple pregnancy rate, duration of pregnancy, birth weight, cumulative incidence of maternal and neonatal complications, and number of embryo transfers to achieve live birth.

A tertiary objective is to compare the rates of pregnancy, pregnancy loss and live birth after the initial embryo transfer between PGS and IVF.

Design and Outcomes

This is a multicenter, randomized trial comparing the efficacy and safety of transfer of single embryo at the blastocyst stage selected by next generation sequencing (NGS) versus conventional morphological criteria. Subjects with 3 or more blastocysts on day 5 of embryo culture will be randomized to the PGS or IVF group. A Freeze-all strategy and a single frozen blastocyst transfer will be performed in both the PGS and IVF groups until all study-specific embryos have been transferred. Subjects in the PGS group will have 3 blastocysts sequenced and euploid embryos will be subsequently transferred one by one. Subjects in the IVF group will have all of their embryos which have reached the blastocyst stage vitri-fied on day 5 or 6. The cumulative live birth rate will be counted after transfers of all euploid embryos in the PGS group and up to 3 blastocysts in the IVF group within 1 year after randomization in both groups if the study-specific embryo transfers are not finished.

The primary outcome is cumulative live birth rate over the (up to) 3 embryo transfers. The secondary outcomes include the Good Birth Outcome rate, cumulative pregnancy rate, cumulative pregnancy loss rate, multiple pregnancy rate, duration of pregnancy, birth weight, cumulative incidence of maternal and neonatal complications (including fetal anomalies) and number of transfers to achieve live birth. The tertiary outcomes are the rates of pregnancy, pregnancy loss and live birth after initial transfer between PGS and IVF.

Interventions and Duration

1. Ovarian hyperstimulation (10 to 30 days): All subjects will receive controlled ovarian hyperstimulation with gonadotropin releasing hormone (GnRH) agonist long or short protocol or GnRH antagonist protocol as per physician preference.
2. Oocyte retrieval, ICSI and embryo culture (5 days): Oocyte retrieval will be performed 34 to 36 hours after oocyte maturation trigger by hCG, GnRHa, or dual trigger. Intracytoplasmic sperm injection (ICSI) will be applied for all included subjects. On the day 5 of embryo culture, women with 3 or more blastocysts will be randomized in a 1:1 ratio into one of two groups: the PGS or the non-PGS group.
3. Embryo biopsy, vitrification and NGS test: Subjects in both groups will have all their embryos frozen and undergo single frozen blastocyst transfer. Subjects assigned to the PGS group will have 3 blastocysts biopsied on day 5 and tested by NGS. Subjects assigned to the non-PGS group will have 3 day-5 blastocysts selected for the first 3 transfers. In both groups, the best blastocycsts by morphologic criteria will be selected for NGS or transfer. Assisted hatching will not be performed.
4. A recovery period after ovarian stimulation of 6-10 weeks to allow, shedding of the endometrial lining, decline of steroid hormones, and restoration of normal ovarian function: Endometrial preparation will be started at the second or third menses cycle after oocyte retrieval. Both natural ovulation cycle and artificial regimen will be allowed for endometrial preparation.
5. Pregnancy evaluation and follow-up (2 weeks to 10 months): All pregnancies will be followed up till termination or delivery.
6. Outcome of subsequent transfers (12-21 months): If live birth isn't achieved by the initial transfer and there are euploid embryos in the PGS group or morphologically transferrable embryos in the IVF group, subsequent single embryo transfer(s) will be performed (up to 3 or up to one year after randomization). The cumulative live birth rate will be counted after transfers of all euploids in the PGS group and 3 blastocysts in the IVF group or all transfers within 1 year after randomization in both groups.

Evaluations

1. Conception: Twelve to fifteen days after embryo transfer, serum Quantitative hCG will be tested. Conception will be diagnosed with a Quantitative hCG of 25 mIU/ml or above.
2. Clinical pregnancy: Twenty days after conception, transvaginal ultrasonography will be performed. Clinical pregnancy will be diagnosed with detection of an intrauterine gestational sac.
3. Obstetric complications, live birth, and neonatal complications including congenital anomalies: These outcomes will be determined with reference to the obstetric and neonatal medical record.

Sample Size and Population

We aim to enroll good-prognosis women who are undergoing their first cycle of IVF. The targeted population is women age 20 to 37y, with 3 or more good-quality blastocysts obtained on day 5 of embryo culture. The exclusion criteria are women with a uterine cavity abnormality or women who plan to undergo preimplantation genetic diagnosis (PGD). Given the likelihood of a high proportion of mosaic embryos in the PGS group and our decision to not transfer mosaic embryos (which are unlikely to achieve live birth and have an unknown risk-benefit ratio), we assume that the cumulative live birth rate after the transfer of PGS-selected euploid blastocysts will unlikely exceed that after serially transferring all the 3 untested blastocysts. This study is therefore designed as a non-superiority study. It is estimated the cumulative live birth rate after 3 single embryo transfers is 65% in each group. To be 80% certain that the upper limit of a one-sided 95% confidence interval (CI) would exclude a difference in favor of the PGS group by more than 7%, 575 patients are required in each group. In consideration of 5% drop-out rate, a total of 1208 subjects will be enrolled. The randomization will be stratified by study site. Blocked randomization will be used with dynamic block size. The random number sequence will be generated by the data coordinating center and will be unknown to all investigators. The randomization will be performed on day 5 of embryo culture via a central online randomization system. At present, 8 centers have volunteered and qualified to participate in subjects' enrollment. It is planned that each center will approximately enroll 80 subjects.

STUDY OBJECTIVES

1.1 Primary Objective

The primary objective is to determine the cumulative live birth rate, defined as delivery of any viable infant at 28 weeks or more of gestation after our interventions. The primary hypothesis is that PGS will result in a non-superior rate of cumulative live birth after up to 3 transfers within 1 year after randomization compared with conventional IVF.

1.2 Secondary Objectives

The secondary outcomes include the Good Birth Outcome rate, cumulative pregnancy rate, cumulative pregnancy loss rate, multiple pregnancy rate, duration of pregnancy, birth weight, cumulative incidence of maternal and neonatal complications (including fetal anomalies) and number of transfers to achieve live birth.
.

The secondary hypotheses are as follows:
1) PGS will be more likely to achieve a Good Birth Outcome than IVF.
2) PGS will reduce the rate of pregnancy loss.
3) The incidence of other maternal and neonatal complications will be comparable between PGS and IVF groups.
4) The rate of multiple pregnancy will be low and comparable between PGS and IVF groups
5) The birth weights will be comparable between babies delivered from PGS and IVF.
6) PGS will reduce the number of embryo transfers to achieve live birth.

1.3 Tertiary Objective

The tertiary objectives are to compare the rates of pregnancy, pregnancy loss and live birth after the initial embryo transfer between PGS and IVF and to compare the cost-effectiveness of PGS and IVF in women with good prognosis.

2 BACKGROUND

2.1 Rationale

The rationale for the study is to establish the risk/benefit ratio of PGS in women with a good prognosis for pregnancy with Level I evidence, as PGS is increasingly being utilized for this indication. Existing trials are underpowered and have not completely tracked the outcomes of public health interest, i.e. live birth, anomalies, and maternal/fetal/infant complications. Additionally they have not examined cumulative results after multiple transfers. The ultimate goal of all infertility treatment, including the use of PGS, is to achieve the live birth of healthy singleton babies without substantial risk to the mother. As the history of PGS is complex, we will discuss more completely in the next section the background and rationale for the study.

We intend to study women with a good prognosis for IVF based on age, i.e. 20-37 years old and an adequate response to ovarian stimulation and good embryo quality, i.e. at least 3 blastocysts obtained. We will study women only during their first IVF cycle to avoid selecting patients at increased risk for implantation failure as well as to exclude patients who may contribute more than one cycle of participation to the study. It has been documented that the rate of euploidy among blastocysts is approximately 60%.[1] We will sequence 3 blastocysts to maximize the chance of obtaining at least one euploid embryo for transfer after PGS testing. Our decision not to perform NGS on all available blastocysts is due to limited study funds. We expect approximately half of subjects in the PGS group will have 2 euploid embryos for transfer. To be comparable between the two treatment groups, we will include 3 transfers of single blastocysts selected by morphological criteria in the non-PGS control group. The cumulative live birth rate will be the primary outcome. We will exclude patients and their husbands with known chromosomal abnormalities by karyotyping. Further we will exclude patients with known uterine or tubal factors which are associated with implantation failure, including uterine congenital malformation, untreated uterine septums, polyps, adenomyosis, submucosal leiomyomas, or hydrosalpinges, or history of intrauterine adhesions (Asherman's syndrome). To minimize the heterogeneity among subjects, those couples who plan to use donated eggs or sperm will be excluded.

2.2 Supporting Data

In vitro fertilization (IVF) is a well-established technology for infertility treatment[2], but IVF does not guarantee success. In the U.S. the live birth rate was 27.3% after fresh embryo transfer and 31.5% after frozen embryo transfer from the ART (Assisted Reproductive Technology) Success Rates tabulated by the Centers for Disease Control and Prevention (CDC) of the United States report in 2013. Traditionally, multiple embryos are transferred to improve pregnancy rates, but this has also resulted in a high rate of iatrogenic multiple pregnancy, which is still one of the major complications of IVF. Elective single embryo transfer (eSET) has been introduced as the most effective way to minimize the multiple pregnancy rate[3,4]. As a consequence, embryo selection becomes crucial to maintain the pregnancy and live birth rates after decreasing the number of embryos transferred. Embryo aneuploidy is thought to be one of the main causes of implantation failure, preimplantation growth arrest, and spontaneous abortion[5-8]. A randomized trial demonstrated that the ongoing pregnancy rate（>24 weeks) was comparable between transferring a single euploid blastocyst and transferring two untested blastocysts[9]. However, conventional morphological score does not predict the genetic conditions of embryos[10].

The embryo aneuploidy rate increases steadily with age[1,11]. However, the embryo aneuploidy rate was also suggested to be high in young patients. Franasiak et al. reported that the aneuploidy rate was 34.15% in patients under 26 years, and 33.99% in patients aged 30 to 38[12]. The average rate of aneuploidy in women under 38 years was 58.9% with blastomere biopsies and 36.1% with blastocyst biopsies[1]. There was also a report that the aneuploidy rate in patients under 35 years was as high as 44.9% (191/425)[13]. PGS is proposed as a potentially effective approach to facilitate embryo selection and eventually improve the singleton live birth rate[4,14,15].

The developmental history of PGS

First generation of PGS (PGS 1.0), which consisted of Day 3 embryo blastomere biopsy, was shown to have no effect, or even a detrimental effect on live birth rate in both young and old women by numer-

ous studies[16-20]. The American Society for Reproductive Medicine (ASRM) and British Fertility Society (BFS), and the European Society for Human Reproduction and Embryology (ESHRE) have issued statements concerning the efficacy and safety of PGS 1.0 [21-23]. All statements expressed that there was insufficient evidence supporting the routine use of PGS1.0. The proposed reasons for ineffectiveness of PGS 1.0 included: First, the embryo damage caused by blastomere biopsy on Day 3 can impact the embryo viability; Second, limited chromosomal testing with fluorescence in situ hybridization (FISH) cannot reflect the chromosomal status of whole genome such that many genetic abnormalities are missed; Third, the insufficient material obtained from Day 3 embryo biopsy may affect the accuracy of diagnosis of mosaicism[24].

PGS 2.0 was developed to screen the genome more thoroughly at a later embryonic development stage, i.e. by performing trophectoderm biopsy at the blastocyst stage. Nowadays, trophectoderm biopsy is commonly used in clinical practice[25,26], and is thought to have no effect on the development and implantation potential of embryos[27]. In addition, more biopsied cells are available with blastocyst biopsy, which increases the accuracy of PGS test and decreases the probability of unrecognized mosaicism[26]. More importantly, it was suggested that after appropriate training, the consistency and reproducibility of results of comprehensive chromosome screening (CCS) were high between different practitioners in different IVF centers [26].

There have been several methods that are used for CCS, i.e. next generation sequencing (NGS), array-comparative genome hybridization (aCGH) and quantitative polymerase chain reaction (qPCR). NGS is the latest approach for CCS with high throughput by parallel analysis of multiple samples in a single sequencing run. NGS is able to detect all types of aneuploidies and can test the nuclear and mitochondrial genomes simultaneously with reduced costs and enhanced precision. Many validation studies have showed that NGS shared a high level of concordance (99-100 %) with array CGH and SNP (single nucleotide polymorphism) array in aneuploidy screening for the same WGA (Whole genome amplification) products[28-32]. In addition, NGS is able to detect lower levels of mosaicism than other methods. *The comparison among different methods for PGS is explained in detail in Supplemental appendix (Table 1).*

Controversial opinions on the application of PGS 2.0

A meta-analysis evaluated three RCTs and eight observational studies comparing PGS and routine embryo morphology selection, the results of clinical implantation rates (IR) and sustained IR beyond 20 weeks showed that PGS can improve IVF outcomes[33]. All patients included in these three RCTs were those with normal ovarian reserve, all had trophectoderm biopsy performed, of which two were analyzed genetically with qPCR, one with aCGH. Of note, our proposed sample size would more than double the cumulative sample size in this meta-analysis. In the observational studies, the targeted patients, embryo stages of biopsy and genetic platforms were all different. These observational studies involved patients with advanced maternal age (AMA), recurrent miscarriage (RM), or recurrent implantation failure (RIF), biopsies on PBs, on day 3 or day 5–6 of embryo development and genetic methods of aCGH, mCGH or qPCR[33]. The conclusion above concurred with those yielded from another two recent systematic reviews of PGS-CCS, by Lee et al and Chen et al. Both analyzed above three RCTs and the other observational studies, and agreed that PGS-CCS is favorable in improving IR and ongoing pregnancy rates in patients with normal ovarian reserve under IVF/ICSI treatments[34,35].

Table 2. Summary of published RCTs regarding to PGS-CCS

Author and year	Treatment groups	Main patient population	Sample Size	Primary Outcome	Live Birth rates	Cumulative live birth rates
Yang 2012[13]	Experimental: Morphological assessment and aCGH-based CCS and fresh SET on Day 6 Control: Morphological assessment and fresh SET on Day 6 Group	Good-prognosis patients(age <35, no prior IVF treatment, no prior miscarriage); **Inclusion criteria:** *a history of regular ovulation; *etiology of infertility was tubal factor or male factor (or both); *no prior IVF treatment had been initiated. *a normal intrauterine contour (confirmed by hysteroscopy), both ovaries intact, basal serum FSH and estradiol on d2-3 at < 10 IU/l and < 60 pg/ml, respectively. **Exlusion criteria:** *IVF patients whose treatment incorporated donor gametes or frozen/thawed embryos were excluded	55 vs.48	Clinical PR: 70.9% vs 45.8%, ongoing PR (>20 wk): 69.1% vs. 41.7%	No	No
Schoolcraft 2012[36]	Experimental: Morphological assessment and fresh blastocyst transfer; Control: SNP-based CCS and frozen SET	AMA（>35）	30 vs.30	ongoing implantation rates: 60.8% vs 40.9%	No	No
Forman 2013[9]	Experimental: qPCR-based CCS and SET Control: Morphological assessment and DET	Normal ovarian reserve(age ≤ 42) **Inclusion** *≤1 previous IVF failure * Maximum prior day 3 follicle stimulation hormone (FSH) level of 12; Minimum anti-mullerian hormone (AMH)of 1.2 within 1 year *Normal uterine cavity demonstrated by saline sonogram, hysterosalpingogram or hysteroscopy within 1 year. *Male partner with greater	89 vs.86	Ongoing PR (>24 wk): 60.7% vs 65.1%	Yes (follow-up) 61% vs 65%	Yes[37] (follow-up through the fresh cycle and up to 1 frozen transfer) 69% vs 72%

Author and year	Treatment groups	Main patient population	Sample Size	Primary Outcome	Live Birth rates	Cumula-tive live birth rates
		than 100,000 total motile spermatozoa. Donor sperm ok.<br>*Body Mass Index (BMI) less than or equal to 30 kg/m2.<br>**Exclusion**<br>*Diagnosis of chronic anovu-lation (cycles typically longer than 90 days)<br>*Diagnosis of endometrial insufficiency- prior cycle with endometrial thickness less than 6mm, abnormal endome-trial echotexture, persistent endometrial fluid.<br>*Clinical indication of aneu-ploid screening (i.e. history of loss of chromosomally abnor-mal pregnancies)<br>Clinical indication for *PGD for single-gene disorder (i.e. PGD is needed to select against the transfer of embryos affected with a specific condi-tion)<br>*Use of testicular aspiration or biopsy procedures to obtain sperm<br>*Unevaluated ovarian mass or surgically confirmed stage IV endometriosis<br>*Presence of hydrosalpinges which communicate with the endometrial cavity<br>*Any contraindication to un-dergoing in vitro fertilization				
Scott 2013[38]	Experimental: qPCR-based CCS and fresh DET on day 6；<br>Control：<br>Fresh DET on Day 5	Normal ovarian reserve(21-42), ≤1 previous IVF failure<br>**Inclusion**<br>*Age of female partner of < 43 years<br>*Normal day-three FSH level (< 15 mIU/mL)<br>*Normal uterine cavity<br>*Sufficient ejaculated sperma-tozoa in male partner for ART<br>*Maximum of one prior failed IVF cycle<br>**Exclusion:**<br>*FSH level ≥ 15 mIU/mL<br>*BMI greater than 32 kg/m2<br>*Contraindication to gonado-tropin stimulation<br>*Unevaluated Ovarian mass<br>*Need for surgical sperm re-moval<br>*Any contraindication to un-	72 vs.83	Clinical IR ： 79.8% vs 63.2%；<br>Sustained IR：66.4% vs 47.9%	Yes(2nd) deliv-ery rate: 84.7% vs 67.5%	No

Author and year	Treatment groups	Main patient population	Sample Size	Primary Outcome	Live Birth rates	Cumula-tive live birth rates
		dergoing in vitro fertilization<br>*Age greater than 43 years<br>*Presence of hydrosalpinges which communicate with the endometrial cavity<br>*Clinical indication for PGD (undergoing IVF with PGD to rule out a known genetic de-fect)				
Munne 2017	Experimental：<br><br>selection by NGS-based PG S<br><br>selection based on morphology	Women（25-40 years）<br><br>Inclusion Criteria: Patient undergoing IVF At least 2 blastocysts suitable for biopsy on day 5 or 6 of embryo devel-opment<br><br>Exclusion Criteria: History of more than two prior implanta-tion failure following IVF<br><br>History of more than one mis-carriage of viable pregnan-cy One or both partners known to be carrier(s) of a chromoso-mal abnormality Known ge-netic carrier couple and/or one or both partners carrier of a known autosomal dominant<br><br>disorder<br><br>Any other non-study related preimplantation genetic testing<br><br>Use of donor oocytes<br><br>Use of gestational carrier (sur-rogate or donor egg recipient).<br><br>Severe oligospermia (<1,000,000 sperm/ml); Surgi-cal Sperm Retrieval for reasons other than post-vasectomy and CAVD<br><br>Low ovarian reserve with (FSH) >10 IU/L on day 2-4 of a prior menstrual cycle and/or (AMH) <7 pmol/L (or <1	274 vs. 314	Ongoing Pregnancy rate<br><br>49.6% vs. 45.9<br><br>50.8% vs. 37.2%(35-40 years)	No	No

Author and year	Treatment groups	Main patient population	Sample Size	Primary Outcome	Live Birth rates	Cumula-tive live birth rates
		ng/ml)<br><br>Gender selection cy-cles Concurrent participation in another clinical trial				

There are also opponents of PGS, who cite several arguments against its use. First, the overall cumulative live birth rate per cycle started can never exceed the serial transfer of all embryos, so PGS may just be a short cut to that same final outcome. Second, the procedures of biopsy and sequencing for PGS generate more expense for the patients. Cost-effectiveness analysis of PGS was performed in the patients with AMA and unexplained RM separately, and it was determined that IVF with PGS was not a cost-effective strategy for increasing live birth[39,40]. Third, the possibility of false positive and false negative results can never be ignored. Many factors can also lead to misdiagnosis in PGS, such as contamination, allele drop-out, preferential amplification and mosaicism of the embryo. While a false positive result may reduce pregnancy outcomes by a missed transfer of a healthy embryo, and a false negative result can lead to poor pregnancy outcomes by the transfer of an abnormal embryo. As described by Greco et al., mosaic embryos can develop into viable euploid newborns[41], and the discarding of these embryos as abnormal ones can also reduce the chance for a live birth. Last, the potential effects of embryo biopsy on extrauterine and intrauterine development of embryos needs to be further investigated in prospective studies with detailed follow-up of pregnancy and neonates to systematically and prospectively collect adverse events.

Kang et al. compared IVF outcomes between frozen euploid blastocysts transfers (n=274) and fresh nonbiopsied blastocyst transfers (n=863) and concluded that although PGS-CCS can improve clinical pregnancy and live birth rates in women >37y, it was ineffective in patients ≤37y [42]. The PGS advantage also disappeared after the data were analyzed per cycle in the >37y age group. Furthermore, a recent reanalysis of U.S. national data in 2011-2012 of 5,471 fresh autologous IVF cycles with PGS and 97,069 cycles without PGS showed that the application of PGS decreased chances of live birth, whereas miscarriage risk was similar. Improvements in live birth and miscarriage rates were reported in the older PGS group (＞37 y), but possibly ascribed to favorable patient selection biases rather than the PGS procedure[43]. Gleicher et al. noted the favorable patient bias in their review, as some patients with AMA and poor ovarian reserve were excluded from analysis as they had no or few embryos reaching blastocyst stage for trophectoderm biopsy and PGS. This dropout will lead to fewer embryos transferred and analysis errors[44]. Additionally, Murugappan et al performed a respectively intent-to-treat analysis of clinical outcomes in 112 RM patients attempting IVF-PGS and 188 RM patients to be expectantly managed and found that pregnancy rate, LBR and clinical miscarriage rate were similar, but median time to pregnancy was longer in IVF-PGS group [45]. Shahine and Lathi highlighted in their review that high-quality studies that identify which patients are most likely to benefit from PGS and include live birth rates per initiated cycles based on "intent-to-treat" are still needed [46].

Limitations of published clinical trials and urgent need of high-quality RCTs

Large heterogeneity exists among these clinical trials, such as different genetic platforms, different biopsy methods and stage[33], and the number of RCTs published so far is rather limited, which undermines the credibility of the conclusions drawn from meta-analyses and systematic reviews.

Most importantly, critics of PGS claimed that the efficacy of published clinical trials is compromised for several important limitations of study quality and design. First, concerns have been expressed about the improper design of RCTs to date, including non-comparable interventions, lack of allocation concealment, large loss to follow-up, and inappropriate outcome measures, for example not reporting live birth rates or perinatal complications[47]. Second, in most published RCTs, only a single transfer cycle was included and the results of subsequent FETs were not counted. Increasingly national ART registries are requiring cumulative live birth rates as the ultimate measure of cycle success to avoid gaming the system which in the past rewarded primarily pregnancies that occurred after the initial fresh transfer. Third, the pregnancy outcomes were analyzed with the unit of per transfer cycle, rather than with per cycle started or per woman with an intent-to-treat principle[35,44,47]. The implantation rate (IR) with denominator of transferred embryos was also inappropriate as the only denominator of interest is the human subject fate, not the embryo's. The outcomes of subsequent FETs were not included in the analysis either. The live birth rate (LBR), especially cumulative LBR following a single oocyte retrieval is the most relevant outcome and the ultimate goal of infertility treatment [47,48]. The application of PGS helps to eliminate non-viable embryos and probably improves IR and clinical pregnancy rate per transfer, but may also lead to a decrease of transfer cycles and transferred embryos at the same time, resulting in a lower LBR per cycle or per women[47]. Fourth, the sample size of published studies was small.

At present, almost all of the ongoing RCTs on comparison of PGS 2.0 and routine IVF treatment listed in *clinicaltrials.gov* still target the IR, or ongoing IR, ongoing pregnancy rate as the primary outcome measure rather than LBR or cumulative LBR, and some RCTs have terminated because of insufficient enrollment. **Registered RCTs registered in** *clinicaltrial.gov* ***are listed in*** **Supplemental appendix (Table 3)**.Therefore there is an urgent need of an adequately powered and designed RCT to clarify the risks and benefits of PGS 2.0.

Rationale for Elective single embryo transfer (eSET)

Current IVF practice and practice guidelines allow for the transfer of multiple embryos in many patients. This strategy is utilized due to the inability to select a single embryo that will lead to a live birth. As the implantation rate continues to improve with the advancement in embryo culture, concerns have increasingly been raised about the risk of multiple pregnancies as the consequence of such a policy and the number of embryos transferred has been reconsidered. By reducing the number of embryos transferred to two, the incidence of high-order pregnancies was dramatically decreased without compromising pregnancy rate;[49] however the perinatal risk of twin pregnancies to both mother and fetuses remains markedly elevated and unchanged with advancing technologies.[50] Thus eSET is the most effective approach to reduce the risk of twin pregnancies and achieve the ideal objective of infertility treatment, i.e. delivery of a healthy term baby after an uncomplicated pregnancy[3,51] which we have termed a Good Birth Outcome in this study. In concert with this, in order to improve the selection of embryos, extended

in vitro culture to blastocyst stage with further determination of the development potential has been increasingly utilized. A blastocyst has greater implantation potential compared with a cleavage-stage embryo.[52] A single blastocyst transfer resulted in significantly higher rates of pregnancy and delivery than a single cleavage-stage embryo transfer.[53] In patients with a good prognosis, it has even been reported that elective single blastocyst transfer results in similar rates of implantation and ongoing pregnancy but a significantly lower rate of twin pregnancies compared with the transfer of two blastocysts.[54] Elective single embryo transfer has been increasingly adopted all over the world, especially in European countries where IVF treatment is covered by the state and closely regulated.[55] Elective single embryo transfer is recommended by ASRM guideline to patients with a good prognosis: age <35 years, more than one top-quality embryo available for transfer, first or second treatment cycle, previous successful IVF, and recipients of embryo from donated eggs.[50] In this study, we will only include subjects aged 20 to 37 years with 3 or more blastocysts in the first IVF cycle. In accordance with the guideline and the goal of avoiding iatrogenic multiple pregnancy, single blastocyst transfer will be performed for both treatment groups.

Rationale for eFET

With the development of technique for embryo freezing, especially the widespread use of vitrification, the implantation rate after frozen embryo transfer has been increasing. Data from SART in 2013 showed the implantation rate after frozen embryo transfer was similar for women under 38 years and higher in women over 38 years than that after fresh embryo transfer. Meanwhile, the supraphysiological levels of steroid hormones during fresh transfer are suspected to effect the endometrial receptivity and the implanting embryo.[56,57] Observational studies showed that singleton pregnancy derived from frozen embryo transfer had lower risk of pregnancy complications than that from fresh embryo transfer.[58] We conducted a multicenter randomized controlled trial in women with PCOS comparing elective frozen embryo transfer with fresh embryo transfer.[59] Our results showed that frozen embryo transfer resulted in a higher rate of live birth and lower rate of pregnancy loss than fresh embryo transfer as well as a higher birth weight after frozen embryo transfer. The complications of pregnancy including twin and singleton were comparable except for a higher risk of pre-eclampsia after frozen embryo transfer versus fresh embryo transfer. This study highlights the need to capture all perinatal complications to fully inform risk-benefit counseling of patients about IVF procedures. Another recent study presented at ASRM this year of fresh vs frozen transfer in patients with unexplained infertility showed comparable ongoing pregnancy rates after fresh or frozen transfer. Thus we believe the current data are favorable supporting elective freezing of embryos having a good prognosis for ongoing pregnancy and/or live birth. At present, for women undergo PGS cycle, embryos are routinely vitrified after biopsy and frozen embryo transfer is performed if euploid embryos are obtained. To keep the two groups in our study comparable and to eliminate the confounder of fresh vs frozen transfer, subjects in the non-PGS control group will also undergo elective frozen embryo transfer.

Rationale for morphologic scoring on embryos

Embryo morphologic assessment is the most commonly used method for embryo selection in the clinical routine. The three-part scoring system for blastocysts was developed in 1999 based on blastocyst expansion, inner cell mass (ICM), and trophectoderm (TE) development[60]. Using this scoring system it was revealed that there was a strong correlation between the clinical outcomes (implantation rates, pregnancy)

and the morphological grade of blastocysts, and the transfer of top-scoring blastocysts produces increased implantation and pregnancy rates [61].

Although this type of selection is simple and direct, there are also many limitations. The main limitation is that it is a static system and can be operator-dependent. More importantly, it is unable to validly reflect the chromosomal status of the embryos. Although there seems to be a relationship between the ploidy status and blastocyst morphology[62], the euploid embryo rate was only 40% -56% in the top-scoring blastocysts[10,62], and miscarriage rate wasn't effectively improved after the transfer of top-quality ones[63]. Thus, morphologic evaluation alone cannot meet the growing demands for embryo selection, especially in the context of SET.

Rationale for tranferring mosaics: Insufficient Data at Present

It has been reported that the majority of human pre-implantation embryos after IVF display chromosome mosaicism, of which the diploid-aneuploid mosaicism is the most common chromosomal constitution[64]. Chromosome mosaicism is most frequently observed in embryos at the early cleavage stages, declining in prevalence as gestation progresses[64,65]. A mouse model of mosaicism revealed that aneuploid cells are progressively depleted from the embryo, consistent with a "clonal depletion" hypothesis, and mosaic euploid–aneuploid embryos have comparable developmental potential to normal embryos, provided they contain a sufficient proportion of euploid cells[66]. A recent observational study showed that mosaic embryos can develop into euploid newborns. Of 18 patients undergoing the transfer of a mosaic embryo, 6 achieved clinical pregnancy and delivered a healthy singleton infant at term[41]. However, the mosaic embryos are usually regarded as abnormal and transfer of mosaic embryos are not recommended in present clinic. Given the limited data about the safety of transferring mosacic embryos, we plan to freeze all mosaic embryos in this study until the risk benefit ratio is clearer.

3 STUDY DESIGN

This is a multicenter, randomized clinical trial comparing the efficacy and safety with transfer of embryos selected by next generation sequencing (NGS) and morphologic criteria versus by morphological criteria alone. Subjects with a good prognosis for IVF success who obtain 3 or more good-quality blastocysts on day 5 of embryo culture will be randomized to the PGS or the non-PGS group. A freeze-all strategy and frozen single blastocyst transfer will be performed in both the PGS and the non-PGS groups. Subjects in the PGS group will have 3 blastocysts sequenced and euploid embryos will be subsequently transferred one by one by FET. Subjects in the IVF group will have three day 5 blastocysts selected for the first 3 transfers based on morphology assessment. The cumulative live birth rate will be counted after transfers of all euploid embryos in the PGS group and 3 blastocysts in the IVF group (up to 3 transfers of single blastocysts in both groups) within 1 year after randomization in both groups.

4 SELECTION AND ENROLLMENT OF SUBJECTS

4.1 Inclusion Criteria

4.1.1 Women who are participating in their first cycle of IVF or ICSI

4.1.2 Women ages 20 to 37 years.

4.1.3 Women who obtain 3 or more good-quality blastocysts defined as morphological score of inner cell mass B or A, trophectoderm C or better, and grade 4 or better on day 5 of embryo culture will be randomized.

4.2 Exclusion Criteria

4.2.1 Women with a uterine cavity abnormality, such as a uterine congenital malformation (uterus unicornate, bicornate, or duplex); untreated uterine septum, adenomyosis, submucous myoma, or endometrial polyp(s); or with history of intrauterine adhesions.

4.2.2 Women who are indicated and planned to undergo PGD, for example, abnormal parental karyotype, diagnosed with monogenic disease, or at high risk for transmitting a genetic disease;

4.2.3 Women who use donated oocytes or sperm to achieve pregnancy;

4.2.4 Women with contraindication for assisted reproductive technology or for pregnancy, such as poorly controlled Type I or Type II diabetes; undiagnosed liver disease or dysfunction (based on serum liver enzyme testing); renal disease or abnormal serum renal function; significant anemia; history of deep venous thrombosis, pulmonary embolus, or cerebrovascular accident; uncontrolled hypertension, known symptomatic heart disease; history of or suspected cervical carcinoma, endometrial carcinoma, or breast carcinoma; undiagnosed vaginal bleeding.

4.3 Study Enrollment Procedures

4.3.1 Recruitment: Potential subjects who meet the preliminary screening criteria (age 20 to 37 years and undergoing their first cycle of IVF) after opting for IVF will be referred to the local investigator before the start of ovarian stimulation by other physicians at study sites. A copy of the informed consent will be given to the interested subjects to read before meeting with local investigators.

4.3.2 Screening log: A standard screening log will be kept by local investigators in each study site to record all the subjects screened (subjects committing to IVF and offered the study), reasons for ineligibility and for nonparticipation of eligible subjects. The local screening log will be reported to DCC monthly.

4.3.3 Consent procedure: The local investigator will explain this trial in detail to potential subjects and their partners and answer any questions raised by them prior to signing the written informed consent to participate in the study. At the start of ovarian stimulation, written consent will be obtained from both the female subject and her partner. A copy of the signed written informed consent form will be given to the subjects.

4.3.4 Assignment: On day 5 of embryo culture, eligible subjects will be randomized via an online central randomization system. The assignment will be informed to subjects and their physicians.

5 STUDY INTERVENTIONS

5.1 Interventions, Administration, and Duration

The study intervention is PGS for embryo selection as compared with conventional morphologic score alone.

Subjects in the PGS group will have 3 blastocysts biopsied on Day 5 and sequenced, which will be selected by morphology score, i.e. the best embryos by morphology will be selected for PGS. Euploids will be transferred one by one by eSET. The selection of embryos for PGS as well as the transfer order of euploid embryos will be decided by morphology score, i.e the best embryos by morphology will be chosen. The other embryos will be frozen according to local routine.

Subjects in the non-PGS group will have 3 day-5 blastocysts selected according to morphologic score for the first 3 transfers. The order of transfer will be determined by morphologic score, i.e. the best embryo will be transferred first. A single frozen blastocyst will be transferred each time. The priority of transfer will be determined by morphology score.

In both groups, if there is a tie between embryos in morphology score on day 5, the day 3 embryo score will be used to select the embryo for PGS and/or transfer.

5.1.1 Blastocyst morphologic score criteria: Gardner criteria [60]

Blastocysts will be graded on a 1 to 6 scale determined by degree of expansion and hatching status, as follows:
Grade 1 (early blastocyst): blastocoele < 1/2 of total embryo volume;
Grade 2 (intermediate blastocyst): blastocoele ≥ 1/2 of total embryo volume;
Grade 3 (full blastocyst): blastocoele fully occupies the embryo;
Grade 4 (expanded blastocyst): blastocoele is larger than early blastocyst and zona pellucida (ZP) demonstrates thinning;
Grade 5 (hatching blastocyst): herniation of trophectoderm cells from the ZP;
Grade 6 (hatched blastocyst): blastocyst has escaped the ZP.

For blastocysts at Grades 3 to 6, the inner cell mass (ICM) and trophectoderm (TE) will also be graded.

The ICM is graded as follows:
A (many ICM cells packed together tightly); B (several ICM cells grouped loosely) and C (very few ICM cells).
TE was graded as follows: A (many TE cells forming multiple epithelial layers); B (few TE cells consisting of a loose epithelium) and C (very few large TE cells).

The pictures of blastocyst grading are found in Figure 2 [67]:

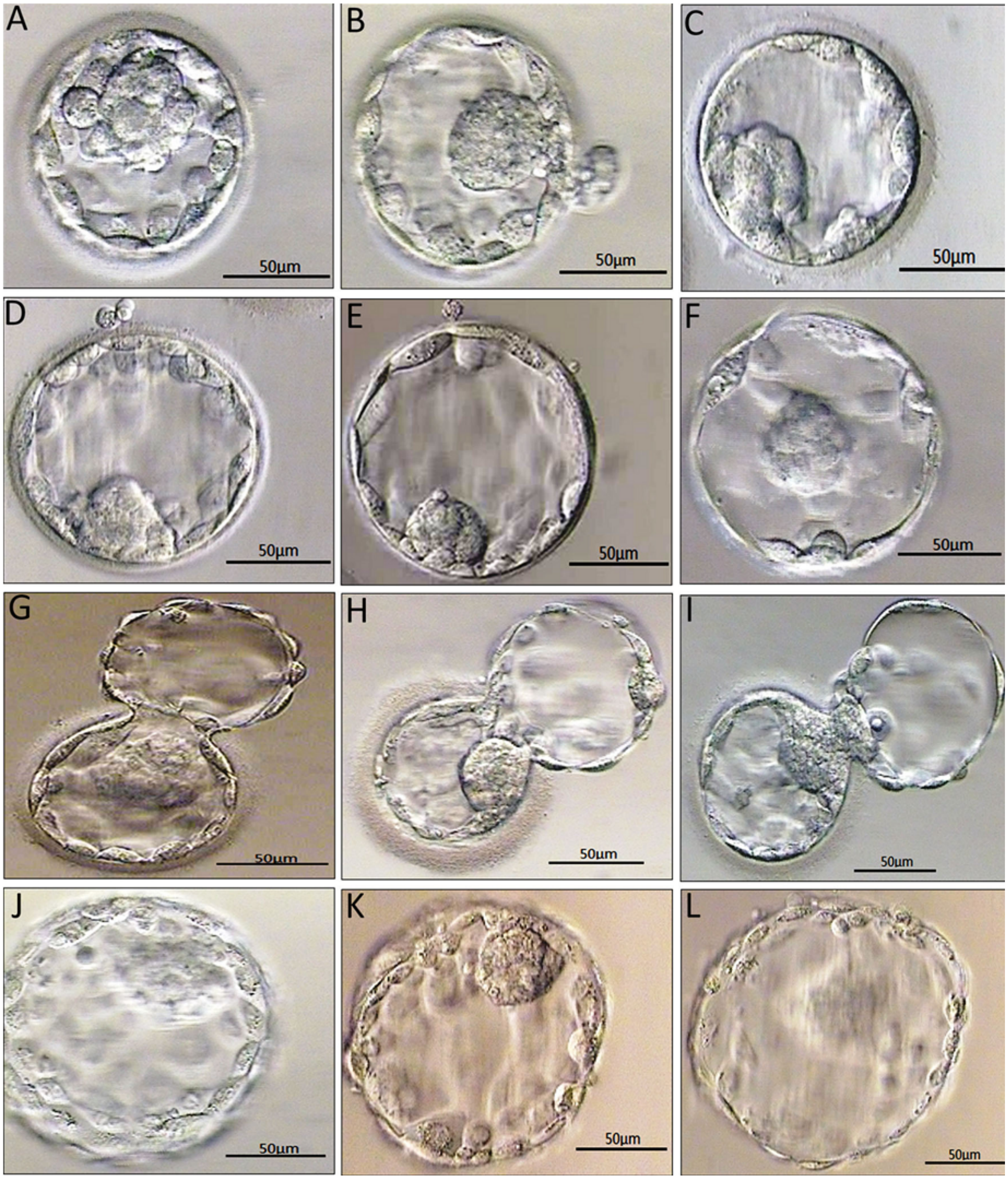

Fig.2 Examples of blastocyst grading: (a)3AA blastocyst; (b) 3AB blastocyst; (c) 3BC blastocyst; (d) 4AB blastocyst; (e) 4BB blastocyst; (f) 4BC blastocyst; (g) 5AA blastocyst; (h) 5AB blastocyst; (i) 5BC blastocyst; (j) 6AA blastocyst; (k) 6AB blastocyst; (l) 6BC blastocyst. Bars=50 µm.

5.1.2 Blastocyst biopsy:

1) Procedure: For blastocysts with score 4BC or better on Day 5 of embryo culture, trophectoderm biopsy will be performed with laser method.

   a) Before the operation, biopsy dishes, polymerase chain reaction (PCR) tubes and fixation slides will be prepared and labeled with patient's name, medical record number and embryo number. A very small channel (10-20 μm) will be opened at the site opposite to the ICM on the zona pellucida of each blastocyst with a diode laser. By entering the zona with the micropipette, 3–5 herniating TE cells will be aspirated with moderate suction, and detached from the blastocyst by synchronously firing several laser pulses at the area of constriction. Next, the aggregate of TE cells will be placed intact into a PCR tube after several washes through hypotonic solution. After the operation, physiological status of the embryo should be observed and recorded.

   b) This operation should be completed by the same embryologist at each study site with more than 3-years experience. Different micromanipulators and different laser frequency used in the participating IVF centers are allowed.

2) Potential side effects: Mechanical damage of embryos, possible impairment of development and implantation potential. It has been reported that mechanical or chemical manipulation of the zona pellucida can increase the risk of monozygotic twinning [68].

5.1.3 NGS tests:

Different NGS platforms (*Illumina MiSeq/HiSeq*、*Life Tech PGM/Proton*) applied in different IVF centers are allowed. The operation procedures will follow the instruction by manufacturers (*Illumina or Life Technologies*).

1) Procedure:
   a) Whole genome amplification (WGA): Blastocysts will be firstly lysed and genomic DNA will be amplified with the routine amplification methods at different sites. Common methods include the Sureplex (Rubicon), multiple displacement amplification (MDA) using Repli-G Midi (Qiagen), and MALBAC (Yikon Genomics). Reagent-positive control (genomic DNA) and negative control (amplification mixture only) are necessary for WGA.

   b) Library preparation: WGA products will be purified and quantified, and subsequently fragmented into a library of small fragments (100 to 200 bp). DNA fragmentation by adding unique adapter sequences will be further performed to simultaneously analyze different blastocyst samples.

   c) Sequencing and sequence analysis: The libraries will be sequenced with paired-end dual indexing using different NGS platforms *(Illumina MiSeq/NextSeq550 or Life Tech PGM/Proton)*. Reads will be aligned to the human genome hg19 and then filtered by removing unmapped reads, duplicate reads and reads with low mapping scores. The following bioinformatics will be accomplished by corresponding software according to the different platforms. Each chromosome will be divided into intervals each approximately

covering 1 Mb of sequence. Filtered reads from each sample will be then mapped into the corresponding chromosome interval or bin. The count data in each bin will be normalized using GC content, and in-silico reference data in order to remove bias. The normalized bin counts will be then re-expressed as copy number by assuming the median autosomal read count corresponds to copy number two. The bin-wise copy number values for each chromosome will be smoothed with a 13-bin sliding median. Automated copy-number status for each chromosome will be determined using the median of smoothed copy-number values across the chromosome as described elsewhere [28].

d) Classification of results: Chromosomal aneuploidies are detected as copy number imbalances. In particular, the analysis pipeline expects a default copy number of 2 for autosomes; the sample sex and sex chromosome copy numbers are determined by an initial calling algorithm. Embryos will be diagnosed as abnormal or aneuploid if the median chromosomal copy number measures deviated from the default copy number. Chromosomal gain or trisomy (copy number >2) and chromosomal loss or monosomy (copy number <2) are seen as horizontal green bars above and below, respectively, the copy number state of 2. Embryos will be diagnosed as normal or euploid if the generated plot showed no gain or loss.

   If amplification failure occurs, the embryo will be regarded as an unknown result and transferred according to the same morphologic grading as the IVF group and analyzed by ITT in the assigned treatment group.

   Mosaic embryos will be frozen and not transferred in this study, until we have further evidence of the safety and efficacy of transferring mosaic embryos. Because the interpretation of an acceptable embryo to transfer is an evolving field, we will modify our policies regarding transferable embryos as new information appears.

2) Potential side effects: Possible existence of false positives and false negatives due to contamination or mosaicism leads to transfer of abnormal embryos or discarding of viable ones. The result is defined as false negative when the actual chromosomal constitution is abnormal or chaotic but the sequencing result is euploid. The result is defined as false positive when the actual chromosomal constitution is euploid but the sequencing result is abnormal or chaotic.

5.2 Handling of Study Interventions

Embryo biopsy and NGS will be performed at local sites.

1) Embryo biopsy should be completed by the same embryologist at each study site, who has more than 3-years experience to minimize damage on embryos, and different micromanipulators and different laser frequency used in the participating IVF centers are allowed. Biopsy should be performed on Day 5 of embryo culture when blastocysts reach score 4BC or better and fully expand. The biopsy site should be right opposite to the ICM and only 3-5 herniating TE cells will be aspirated. After the operation, survival

of the embryo is regarded as a success and operation time should be recorded. Patient's name, the medical record number and embryo number should be clearly marked to ensure the consistency.

2) Different NGS platforms *(Illumina MiSeq/NextSeq550 or Life Tech PGM/Proton)* applied in different IVF centers are allowed. This reflects current practice where different platforms are used. The resolution value is defined as 4Mb. For laboratory environment and facilities, reagent preparation district, WGA preparation district, library preparation district, product purification district and sequencing district should be separated to prevent contamination and buffering area should be set between the adjacent districts. For laboratory technicians, professional training and technical competence is required. For WGA, first, sample-positive control and negative control are necessary; Second, concentration of products detected using Qubit assay should be no less than 20ng/μl, and those of negative control should be less than 10ng/μl; Third, two technicians are needed to prevent confusion when to transfer samples between tubes. For library preparation, the concentration should be more than 0.5 ng/μl and the volume should be no less than 20μl. For sequencing results, the quality control standard of different platforms should be established and complied with. For example, based on Proton of Life Tech, the loading rate should be more than 60%, polyclone rate no more than 40%, low quality rate no more than 30%, and reads counts of each sample larger than 1M. In the last, all operations should be completed by two technicians together in the lab and auditors should audit the original data and check the sequencing report.

This is an open-label unblinded trial because it is not possible to mask the embryo biopsy.

5.3 Concomitant Interventions

5.3.1 Required Interventions

Participants will receive controlled ovarian hyperstimulation (COH) with agonist via a long or short protocol, or an antagonist protocol, HCG or GnRHa trigger final oocyte maturation, and standard ultrasound guided oocyte retrieval, ICSI procedure and embryo culture according to clinical routine in different study sites. An elective freeze-all strategy and single frozen embryo transfers will be employed. The transfer order in the PGS group will be decided by both NGS results and embryo morphologic score, while transfer order in the non-PGS group will be decided by morphologic score alone. The endometrium for FET will be prepared either through a natural ovulation cycle or artificial cycle. The choice of which will be up to the site investigator. Luteal phase support will be administrated according to the routine in different sites. A pregnancy test will be performed 2 weeks after embryo transfer. If pregnancy is confirmed, luteal phase support will be continued till 10 weeks of gestation. The follow-up will continue until termination or delivery.

1) COH and oocyte retrieval:

GnRH agonist long or short or antagonist protocol will be allowed for ovarian stimulation at the discretion of local investigators. The long protocol starts with GnRH agonist (triptorelin) administrated in the mid luteal phase of the previous cycle and gonadotropin is initiated when satisfactory pituitary desensitization is achieved. The short protocol starts with administration of GnRH agonist on day 2 or 3 of the menses and gonadotropin 2 days later. The antagonist protocol starts with admin-

istration of r-FSH on day 2 or 3 of the menses cycle and combines with antagonist (cetrorelix or ganirelix) on day 5 or 6 after gonadotropin initiation or when dominant follicles ⩾12 mm mean diameter, and continues until the trigger day. Then the r-FSH dosage and addition of other drugs (HMG, recombinant growth hormone et al.) will be adjusted in the light of follicle growth monitored by transvaginal ultrasound and serum steroid levels.

HCG and/or GnRHa trigger for final oocyte maturation will be administrated when at least two follicles ⩾18 mm mean diameter. Transvaginal ultrasound guided oocyte retrieval will be performed 34 to 36 hours after trigger.

2) ICSI procedure, embryo culture, and embryo score

The oocytes will be inseminated approximately 4~6 hours after follicular aspiration. Fertilization will be achieved by ICSI in all subjects. All embryos will be cultured in the sequential media to blastocyst stage. Embryo score by Gardner criteria. Subjects with 3 or more blastocysts with score 4BC or better on day 5 will be randomized to PGS or non-PGS group.

3) Embryo vitrification

All embryos will be frozen and no fresh embryo will be transferred.

4) Endometrial preparation, embryo transfer and luteal phase support

Endometrium preparation will performed with either a natural ovulation cycle or an artificial regimen depending on the choice of the site investigators after the second spontaneous menses following oocyte retrieval. A single frozen blastocyst will be transferred each time. Luteal phase support will be added according to local routine. The procedure will be completed under transabdominal ultrasound guidance (with full bladder). All medications used for endometrial preparation and luteal phase support will be documented.

If the viability of the scheduled embryo is severely impaired or lost after thaw, the next one will follow; and if all study-specific embryos (up to N = 3) in both groups lose viability, the surplus embryos will be thawed subsequently for detection or transfer, however, such embryo transfers will not be included in this study.

5) Pregnancy evaluation and follow-up

Serum quantitative hCG will be measured to determine pregnancy 2 weeks after embryo transfer. If a biochemical pregnancy has been achieved, transvaginal ultrasound scan will be performed 35 days after embryo transfer to evaluate for clinical pregnancy. If a clinical pregnancy has been achieved, ultrasound scan will be repeated at 11 weeks gestation to confirm ongoing pregnancy. The subsequent follow-ups will be implemented every three months and continue until termination or delivery.

The cumulative pregnancy outcomes will be followed up after transfers of all euploid embryos in the PGS group and up to 3 blastocysts in the IVF group within 1 year after randomization in both groups if the study-specific embryo transfers are not finished.

Nonviable pregnancy is defined as a crown-rump length ≥7 mm but no heartbeat or mean sac diameter ≥25 mm but no embryo or absence of embryo with heartbeat 11 days or 2 weeks later after a gestational sac is detected with ultrasound scan. Complete spontaneous miscarriage refers to complete spontaneous pregnancy loss without curettage or medical abortion after a clinical pregnancy is confirmed. For women who end up with pregnancy loss, subsequent frozen embryo transfer will be scheduled 3 months later if there are study-specific blastocysts left.

5.3.2 Prohibited Interventions

Couples who plan to use donor eggs or sperm to become pregnant will be excluded from this study.

5.3.3 Precautionary Interventions

We do not anticipate at this time any modifications to the study interventions

5.1.4 Adherence Assessment

In this trial, the study intervention (IVF with or without PGS) will be performed by local embryologists in laboratory. Adherence is expected to be high. No special assessment is planned in this trial. From our previous experience, subjects may choose or doctors may recommend alternate forms of IVF treatment after randomization, and they will be free to do this. We will track this and anticipate it will occur infrequently (<5% of subjects). If deviations or violations from protocol occur, they will be recorded by standard forms.

6 CLINICAL AND LABORATORY EVALUATIONS

6.1 Schedule of Evaluations (Table 4)

**Evaluation**	**Screening**	**Day 3 of embryo culture**	**Day 5 of embryo culture**	**2 wk after ET**	**5 wk after ET**	**10 wk after ET**	**28 weeks gestation**	**37 weeks gestation**	**Delivery**	**6 weeks after delivery**	**1 year after randomization**
Informed Consent	X										
Documentation of indication for IVF or ICSI	X										
Medical/Treatment History¶	X										
Physical Exam*	X										
Ultrasonography	X				X	X					
Basal steroid hormone, AMH	X										
Safety tests (CBC, liver and renal function, coagulation test, HBV, HCV, HIV, syphilis, TORCH, urinalysis)	X										
Randomization to Study Group			X								
Pregnancy Test				X							
Stored blood sample	X					X					
Telephone follow-up							X	X		X	X
Questionnaires※	X										X

¶Previous illness history including hypertension, thyroid diseases, diabetes and cancer will be reviewed. Menstrual and reproductive history as well as infertility history will also be reviewed.

*Physical exam will be performed for both female and male. Height, weight, and blood pressure will be measured.

※Ferti QoL will be completed at baseline.

6.2 Timing of Evaluations

6.2.1 Pre-Randomization Evaluations

These evaluations occur prior to the start of ovarian stimulation.

Screening

The goal of screening will be to exclude established factors affecting embryo implantation (i.e. uterine abnormality, etc.) or major medical illness. Screening will be performed in women who are indicated for IVF or ICSI to become pregnant. Women ages 20 to 37 who are interested in this trial and about to undergo their first cycle of IVF or ICSI will be referred to local co-investigators for screening. After the couple signs informed consent, medical history, physical examination, ultrasonography and laboratory tests will be obtained or performed. Karyotyping will be carried in all potential subjects (both female subjects and male partners) to exclude those who need PGD as advised by genetic counselling. This is done routinely in China. Hysteroscopy will be performed when a uterine abnormality is suspected by ultrasonography. All tests relating to data that will likely be part of the baseline data or final analyses (basal steroid hormone levels, ultrasound, and semen analysis) should be performed within 3 months before starting ovarian stimulation and should be performed at nationally qualified labs. During IVF procedure and pregnancy follow-up, all tests and evaluations will be run at local study sites as part of clinical evaluation.

Pre-Entry

During ovarian stimulation, ultrasonography and serum steroid hormone assays will be performed at the local study site. Ovarian hyperstimulation syndrome as well as other adverse events will be recorded. Subjects whose cycles are cancelled, who have no oocytes, or who experience no fertilization will not be eligible for randomization. On day 3 of embryo culture, embryo number and morphological score will be recorded. On day 5 of embryo culture, embryo number and morphological score will be recorded again. Women with less than 3 good-quality blastocysts will not be randomized.

Randomization

On day 5 of embryo culture, women with 3 or more good-quality blastocysts will be randomized into the PGS or the non PGS group.

6.2.2 On-Study/On-Intervention Evaluations

For women assigned to the PGS group, the result of NGS for the sequenced 3 blastocysts will be recorded. The regimen used for endometrial preparation (natural ovulation cycle or artificial cycle) will be recorded. The endometrial thickness and blood flow index on day of ovulation or initiation of progestin will be recorded. Adverse events will be inquired and recorded at every visit.

6.2.3 Intervention Discontinuation Evaluations

After randomization, further participation in the study will be discontinued in the following situations: a) no euploid embryo is obtained in patients assigned to the PGS group after NGS test; b) Subjects who run out of the protocol-specified embryos; c) Subjects who do not complete transfers of the protocol-specified embryos within 1 year after randomization. Subjects who conceive will be followed to the termination of the pregnancy.

On Study/Off-Intervention Evaluations

Two weeks after embryo transfer, serum hCG will be measured. Conception will be diagnosed if HCG level ≥ 25 mIU/mL. Five weeks after embryo transfer, transvaginal ultrasound scan will be performed. Clinical pregnancy will be diagnosed if intrauterine gestational sac is detected. Ten weeks after embryo transfer, ultrasound scan will be repeated. Ongoing pregnancy will be diagnosed with evidence of fetal heartbeat by ultrasound. Adverse events will be inquired and recorded at every visit. We will follow up with the patient during and after pregnancy to obtain all outcomes including copies of the medical records of the pregnancy and where applicable, neonate.

6.2.4 Final On-Study Evaluations

After delivery, obstetric and neonatal records will be collected. The obstetric and perinatal complications including congenital anomalies and birth weight will be recorded. We will also contact subjects by telephone as noted above.

6.2.5 Off-Study Requirements

At 6 weeks after delivery, subjects will be followed up by telephone call to collect the puerperal and neonatal complications.

6.3 Special Instructions and Definitions of Evaluations

6.3.1 Informed Consent

Subjects who are interested in this study and pass pre-screening (i.e. first cycle of IVF or ICSI, age 20 to 37, etc.) will be referred to local co-investigators for comprehensive screening. On screening visit, the study will be explained to couples in detail and all questions answered prior to signing written informed consent to participate in the study. The husband will also be required to sign a consent form at the time of the screening visit. The principle investigator at study sites will not be the primary caregiver for study participants. The couple-signed consent will be kept together with their case report forms. One copy of the signed consent will be kept by subjects. We will also obtain medical and reproductive histories and a QOL questionnaire (Ferti-QOL) at this visit.

6.3.2 Documentation of indication for IVF

The indications for IVF are as following: a) tubal factor determined by hysterosalpingogram, laparoscopic inspection, or sonohysterogram; b) ovulation dysfunction and failed to become pregnant with ovulation induction; c) endometriosis; d) male factor; e) unexplained infertility. We will track the reason for IVF in all couples.

All couples will undergo karyotyping prior to IVF as is routine in China. Couples with parental karyotypes that show a balanced translocation, Robertsonian translocation, some types of inversion or monogenic disease will be suggested to undergo PGD and excluded from this study.

6.3.3 Medical/Treatment History

The comprehensive medical history questionnaire including questions about medical health, reproductive history, infertility and treatment history, surgery history, and family history will be completed.

6.3.4 Physical exam

A physical exam with a standard pelvic exam will be performed on all patients by a study physician. Height and weight will be recorded to the nearest 0.1 cm, 0.1 kg. Participants will be weighed while dressed in light clothing, without shoes. Blood pressure will be determined in the right arm in the sitting position. Elevated blood pressures (≥140/90) will be repeated following acclimation to the study environment. Pap smear and/or HPV test as needed will performed as recommended by 2015 ACOG guidelines. Patients with cytological abnormalities will need to have these resolved prior to study entry.

6.3.5 Transvaginal ultrasonography

An ultrasound exam will be performed with a transvaginal probe. The following measures will be obtained: uterine dimensions, leiomyoma presence and size, other uterine abnormalities, endometrial thickness, ovarian size in three dimensions, the size of the largest ovarian follicle, and antral follicle count (all follicles <10 mm diameter on ultrasound exam).

6.3.6 Laboratory Evaluations

Blood work including baseline assessment, follicles development monitoring, and pregnancy tests will be run in the local lab. The baseline steroid hormone measurements (FSH, LH, E2, T, PRL, TSH) will be performed at day 2 to 4 of menstrual cycle (spontaneous or progestin-induced). Embryo biopsy and NGS will be done in the local embryo lab by staff at each site proficient in the use of this technique.

6.3.7 Pregnancy test

Serum β-HCG will be measured 2 weeks after embryo transfer. The cutoff to diagnose conception will be 25 mIU/mL.

Transvaginal ultrasound will be performed 35 days after embryo transfer to determine location and viability of the pregnancy. If no original heart beat is detected in the gestational sac, ultrasound scan will be repeated 11 to 14 days later. If original heat beat does not appear then, inviable pregnancy will be diagnosed and curettage will be performed. Subsequent frozen embryo transfer will be scheduled 3 months later if there are study-specific blastocysts left.

Transvaginal ultrasound will be repeated at 11 to 12 weeks gestation to diagnose ongoing pregnancy. Subjects with fetal heart beat disappeared will be suggested to undergo curettage. Subsequent frozen embryo transfer will be scheduled 3 months later if there are study-specific blastocysts left. Subjects with ongoing pregnancy will be referred to their preferred obstetricians.

All pregnancies will be followed up till termination or delivery. At delivery, obstetric and neonatal record will be collected. The obstetric and perinatal complications and birth weight will be recorded.

6.3.8 Blood sample bank

Serum samples and whole blood sample of female and male will be collected for storage and eventual DNA extraction at the baseline visit. We anticipate that the clinical sites will collect the serum, separate into 5 to 10 1cc aliquots in cryovials, labeled with study ID, sample type, the date of draw and a unique identifier (in the form of a freezer safe barcode label). The serum, blood clots and whole blood will be stored at -20 ℃ or -80 ℃ at the clinical sites until they are transferred to the

repository site.

6.3.9 Questionnaires

Quality of life will be assessed by Ferti QOL international 2008 (simplified Chinese version) at baseline and at one year after randomization. Both male and female will be required to fill in the FertiQoL at these two time points.

7 MANAGEMENT OF ADVERSE EXPERIENCES

7.1 Expected adverse experience for each study intervention (Table 5)

Procedures and events	Discomforts and risks
Controlled ovarian hyperstimulation (COH)	frequent subcutaneous injection, frequent venipuncture, frequent transvaginal ultrasound scan<br>supra-physiologic estrodiol may increase risk of blood clotting or long term possibly certain cancers<br>Ovarian torsion or rupture of ovarian cyst(s)
Ovarian hyperstimulation syndrome (OHSS)	Massive enlargement of ovaries, ascites, i.e.fluid in abdominal cavity, bloating, nausea, vomiting. Severe cases may have fluid in thoracic cavity, breathing difficulties, oliguria even anuria, abnormal liver function, and may require hospitalization, medication or puncture drainage of fluid in abdomen or thorax. Very severe case may suffer from thrombosis, damage to liver or renal function, even death
Oocyte retrieval	Anesthesia complications, pelvic organ injury, intra-abdominal hemorrhage, puncture site hemorrhage, in serious case surgery or transfusion may be needed, infection and thrombosis
ICSI	Microinjection may injure oocyte, pass unknown disease gene to next generation
Embryo biopsy	Damage to embryo development potential, increased risk of monozygotic twinning
Embryos transfer	Infection
Embryo frozen and thaw	Cessastion of embryonic development, failure of embryo to survive freeze thaw process. The survival rate of thawed blastocysts is 95%.
Standard venipuncture for blood work	Slight pain, bruising at the site of puncture, infection or bleeding at the site
Transvaginal ultrasound	Abdominal or pelvic discomfort
Ectopic pregnancy	May require medicine or surgery treatment, in severe case

	pregnancy site rupture resulting in intra-abdominal hemorrhage, urgent surgery, blood transfusion, even shock or death if treatment delayed

7.2 Definition of adverse events and serious adverse events

Adverse event means any untoward or unfavorable medical occurrence associated with the subject's participation in the research, whether or not considered related to the study intervention. Adverse events can be any of the following:

1. Physical signs or symptoms including medication side effects
2. Abnormal laboratory values
3. Changes in vital signs, physical exam findings, or test results
4. An increase in the frequency or intensity (worsening) of a condition or illness that was present before study enrollment.

Serious adverse event (SAE): Any event temporally associated with the subject's participation in research that meets any of the following criteria:

1. Death;
2. Life-threatening (at immediate risk of death);
3. Severely or permanently disabling;
4. Requires in-patient hospitalization or prolongation of existing hospitalization;
5. Pregnancy loss after 20 weeks gestation;
6. Neonatal death up to 6 weeks after delivery;
7. Results in a congenital anomaly/birth defect;
8. Or any event so deemed as serious by the PI at the site.

Note: A "severe" adverse event is not the same as a "serious adverse event" or SAE. Severity is based on the intensity of the event, whereas seriousness is based upon the event outcome as it poses a threat to the patient's life or functioning.

Unexpected, adverse event: An adverse event is considered "unexpected" if it is not listed in the general investigational plan or protocol; or is not listed at the specificity or severity that has been previously observed and/or specified.

7.3 Recording of adverse event

All observed or volunteered adverse events (serious or non-serious) and abnormal test findings, regardless of study group or suspected causal relationship to the study intervention(s) will be recorded in the subjects' case report form. Report a diagnosis rather than a symptom. For all adverse events, sufficient information will be pursued and/or obtained so as to permit 1) an adequate determination of the outcome of the event (i.e., whether the event should be classified as a serious adverse event) and; 2) an assessment of the casual relationship between the adverse event and the study intervention(s). Continue to follow each event until it is resolved, stabilizes or until 7 days after the last patient enrolled completes follow-up. Adverse events or abnormal test findings felt to be associated with the study inter-

vention(s) will be followed until the event (or its sequelae) or the abnormal test finding resolves or stabilizes at a level acceptable to the principal investigator.

7.4 Causality and severity assessment

The principal investigator will promptly review documented adverse events and abnormal test findings to determine 1) if the abnormal test finding should be classified as an adverse event; 2) if there is a reasonable possibility that the adverse event was caused by the study intervention(s); and 3) if the adverse event meets the criteria for a *serious adverse event*. The situation surrounding the event should be assessed to determine whether it is related to the study. Record the causality of each event as "possibly related" or "not possibly related". If causality is unknown, it should be considered "possibly related".

The maximum intensity of each adverse event should be evaluated and reported as one of the following:

- Mild: events may or may not be volunteered by the patient. The patient is aware of the event, but it is easily tolerated.
- Moderate: signifies discomfort sufficient to interfere with normal activities. A change in therapy may or may not be indicated.
- Severe: side effects are almost always brought up by the patient. These side effects interfere with daily activities and usually require medical intervention.

8 CRITERIA FOR INTERVENTION DISCONTINUATION

List criteria for discontinuing intervention and methods for determining when criteria are met. Include procedures for maintaining subject participation in followup activities.

The intervention is the performance of PGS on up to 3 blastocysts. It will be discontinued at patient or physician request if the patient is assigned to the PGS group.

9 STATISTICAL CONSIDERATIONS

9.1 General Design Issues

This is a multicenter randomized controlled trial comparing the benefit and safety of PGS and conventional IVF in women with good prognosis. Subjects will be randomly assigned to 2 parallel groups with 1:1 ratio. This study is designed as a non-superiority study to test the following primary and secondary hypothesis.

a) The primary hypothesis is that PGS will result in a similar cumulative live birth after up to 3 transfers within 1 year after randomization compared with conventional IVF.
b) The secondary hypotheses are as follows:
    1) PGS will be more likely to yield a Good Birth Outcome.

2) PGS will reduce the rate of pregnancy loss.
3) The incidence of other maternal and neonatal complications will be comparable between PGS and IVF groups.
4) The rate of multiple pregnancy will be comparable between PGS and IVF groups
5) The birth weight will be comparable between babies resulted from PGS and IVF.
6) PGS will require fewer embryo transfers before achieving live birth.

9.2 Outcomes

9.2.1 Primary outcome

The primary outcome is the cumulative live birth rate over the (up to) 3 embryo transfers. Live birth is defined as the delivery of any viable infant at 28 weeks or more of gestation after our interventions, and cumulative live birth rate is calculated by dividing the number of women achieving live birth after transfers of all euploid embryos in the PGS group or 3 blastocysts in the IVF group within 1 year after randomization, by the total number of women randomized to the specific group.

9.2.2 Secondary outcomes:

The secondary outcomes include the Good Birth Outcome rate (defined as a live birth of an infant born at ≥ 37 weeks, with a birth weight between 2500 and 4000g and without a major congenital anomaly), cumulative pregnancy rate, cumulative pregnancy loss rate, multiple pregnancy rate, duration of pregnancy, birth weight, cumulative incidence of maternal and neonatal complications, and number of embryo transfers to achieve live birth.

9.2.3 Tertiary outcomes:

The rates of pregnancy, pregnancy loss and live birth after the initial embryo transfer between PGS and IVF.

9.3 Sample Size and Accrual

Given the possibility of false positive and the decision of not transferring mosaic embryos which are probably to develop into live birth, we assume that the cumulative live birth rate after transferring PGS-selected euploid embryos will be similar with that after serially transferring all the 3 untested blastocysts. This study is designed as a non-superiority study. It is estimated the cumulative live birth rate after 3 single embryo transfers is 65% in the IVF control group. We set a non-superiority margin at an absolute difference of 7% to retain approximately 90% of the clinical effect of the control group. To be 80% certain that the upper limit of a one-sided 95% confidence interval (CI) of absolute difference between groups to reject null hypothesis that PGS is superior to conventional IVF, 575 pa-

tients were required in each group. In consideration of 5% drop-out rate, a total of 1208 subjects will be enrolled. At present, 8 centers have volunteered and qualified to participate in subjects' enrollment. It is planned that each center will approximately enroll 150 subjects.

9.4 Data Monitoring

We will establish an independent Data and Safety Monitoring Board (DSMB) to review and interpret data generated from the study and to review revisions of the protocol prior to their implementation. Its primary objectives are to ensure the safety of study subjects and the integrity of the research data. The DSMB advises on research design issues, data quality and analysis, and research participant protections for the study. The DSMB will hold regular conference calls in English to review the protocol with respect to ethical and safety standards, monitor the safety of the trials, monitor the integrity of the data with respect to original study design, and provide advice on study conduct. The DSMB will review the progress of the trial, adjudicate adverse events, and decide on any premature closure of the study. The call will be coordinated by the DCC who will provide study updates prior to the call via email.

9.5 Data Analyses

The primary analysis will be performed in accordance with the principle of intent-to-treat. Continuous variables will be described as mean ± standard deviation for the normally distributed variables and as median and range for the non-normally distributed variables. Category variables will be presented as frequency and percentage. The differences in the cumulative live birth rate in the two treatment arms will compared by absolute risk difference and its 95% confidence interval (CI). The relative risk and its 95% CI will also be calculated. Non-superiority of PGS to IVF will be considered to be established if the upper limit of the 95% CI of absolute risk difference was shown to lie below the non-superiority margin of 7%.

The differences in the secondary outcomes such as rate of Good Birth Outcome, cumulative pregnancy rate, cumulative pregnancy loss rate, multiple pregnancy rate obstetric and neonatal complications will be tested by Person chi-square analyses. The absolute difference and 95%CI as well as relative risk and 95% CI will also be calculated. The difference between duration of pregnancy, birth weight and times of embryo transfer will be tested by student t test and non-parameter test if necessary.

The tertiary outcomes such as the rates of pregnancy, pregnancy loss and live birth after the first transfer will be analyzed with Pearson chi-square test. Cost-effectiveness analysis will be done by decision analytic model with TreeAge Pro 2014 (2014 Version; TreeAge

software). Cost effectiveness is defined as an incremental cost effectiveness ratio (ICER).The ICER for live birth was calculated as the difference in cost between two treatment groups divided by the difference in live birth rates between the two groups. The secondary analysis will be performed according to per-protocol analyses. The primary and secondary outcomes will be compared according to actual treatment that subjects received and among subjects who complete embryo transfer(s).
Subgroup analyses will be performed according to different stimulation protocol (e.g. agonist long, agonist short, and antagonist protocol), different endometrial preparation protocol for FET (e.g. natural ovulation cycle and artificial cycle), age groups (e.g. 20~30 group, 30~35 group and >35 group), with or without history of recurrent spontaneous abortion.
Any deviations from the previously described statistical plan will be described and justified in a protocol amendment.

10 DATA COLLECTION, SITE MONITORING, AND ADVERSE EXPERIENCE REPORTING

10.1 Records to Be Kept

The baseline characteristics of subject, outcome of ovarian stimulation and in-vitro fertilization, and pregnancy outcome will be collected with standard case report form (CRF) by local investigators. The hard copy of CRF will be kept in study site in a study-specific cabinet with lock. For the purpose of managing the data, monitoring the process and promoting the transparency of the study, a web based database, Clinical Trial Electronic Case Record Form (eCRF system) (http://www.clinicaltrialecrf.org), will be used to record and deposit the patients' data and results. The collected data will be input into this online database which can only be accessed by authorized personnel.

10.2 Role of Data Management

10.2.1 *Clinical site responsibilities in data collection and management.*

Local investigators or assigned coordinator are responsible to timely collect the data as required by CRF. Effort should be made to minimize missing data and inaccurate data. All missing data must be explained. If a space on the CRF is left blank because the procedure was not done or the question was not asked, write "N/D". If the item is not applicable to the individual case, write "N/A". If any entry error has been made, to correct such an error, draw a single straight line through the incorrect entry and enter the correct data above it. All such changes must be initialed and dated. DO NOT ERASE OR WHITE OUT ERRORS. They are also responsible to input the collected data into electronic database. The data collected in CRF will be de-identified. Local investigator or coordinator will be responsible to keep the confidential of the data collected.

10.2.2 *DCC responsibilities in data management.*

The data coordinator center (DCC) will conduct monthly comprehensive data checks, as well as regular manual checks (within the database system). Manual checks will identify more complicated and less common errors. The data manager will query sites until each irregularity is resolved. The data coordinator center will also responsible for the database clean-up and final data analysis.

10.3 Quality Assurance

Quality control of data will be handled at three different levels. The first level is the real-time logical and range checking built into the web-based data entry system. The research coordinators and data entry clerks at the participating sites are required to ensure the data accuracy as the first defense. The second is the remote data monitoring and validation that is the primary responsibility of the data manager and programmer at the DCC. The data manager will conduct monthly comprehensive data checks, as well as regular manual checks (within the database system). Manual checks will identify more complicated and less common errors. The data manager will query sites until each irregularity is resolved. The third level of quality control will be the site visits, where data in our database will be compared against source documents. Identified errors will be resolved between the DCC and clinical sites. The visits will assure data quality and patient protection.

10.4 Adverse Experience Reporting

The site PI will report the SAE by completing and signing the Serious Adverse Event Report Form within 24 hours of discovery, and then emailing document in PDF format to the protocol PI. The site PI must determine and record on the SAE form whether the SAE is unanticipated or anticipated, and if it is related, possibly related, or unrelated to participation in the research. Then the protocol PI will determine whether the SAE need to be reported to DCC or not. If DCC has been reported to, DCC staff will enter the SAE information in the central database and report to DSMB. Upon receiving notification of an SAE, the DSMB will review it via a closed-session email or conference-call discussion. Then DSMB will send a report to DCC within two weeks; reports for life-threatening SAEs will be submitted in one week. The DSMB report will include: statement indicating what related information the DSMB reviewed; the review date; the DSMB's assessment of the information reviewed; and the DSMB's recommendation, if any, for the DCC. The DCC will forward reportable events to protocol PI and all investigators. The Protocol PI will evaluate the frequency and severity of the SAEs and determine if modifications to the protocol and consent form are required. Site PIs will report the SAE to their site IRB according to local IRB requirements.

Responsibilities of PI at Site of SAE:

1. Site PI should determine and sign on the SAE form if the adverse event is: Unanticipated or Anticipated Related, possibly related or unrelated to participation in the research
2. Reporting is done by submitting the SAE form with the PI's signature in PDF format to the Protocol Leader via email.
3. Reporting timeline:
   i. Unanticipated and related/possibly related SAE must be reported to the protocol PI within 1 business day of discovery.
   ii. Anticipated and related/possibly related SAE must be reported to the protocol PI within 5 business days of discovery.
   iii. Unrelated SAE (anticipated or unanticipated) must be reported to the protocol PI within 10 business days (no more than 3 weeks) of discovery.
4. If the SAE is ongoing, the site PI will send follow-up reports to the protocol PI until the SAE is resolved.

11 HUMAN SUBJECTS

Institutional Review Board (IRB) Review and Informed Consent

This protocol and the informed consent document and any subsequent modifications will be reviewed and approved by the IRB or ethics committee responsible for oversight of the study. A signed consent form will be obtained from the subject. The consent form will describe the purpose of the study, the procedures to be followed, and the risks and benefits of participation. A copy of the consent form will be given to the subject.

Subject Confidentiality

All laboratory specimens, evaluation forms, reports, video recordings, and other records that leave the site will be identified only by the Study Identification Number (SID) to maintain subject confidentiality. All records will be kept in a locked file cabinet. All computer entry and networking programs will be done using SIDs only. Clinical information will not be released without written permission of the subject, except as necessary for monitoring by IRB.

Study Modification/Discontinuation

The study may be modified or discontinued at any time by the IRB, or DSMB as part of their duties to ensure that research subjects are protected.

12 PUBLICATION OF RESEARCH FINDINGS

Publication of the results of this trial will be governed by the policies and procedures developed by the Steering Committee. Any presentation, abstract, or manuscript will be made available for review by steering committee prior to submission.

13 REFERENCES

1. Harton GL, Munne S, Surrey M, et al. Diminished effect of maternal age on implantation after preimplantation genetic diagnosis with array comparative genomic hybridization. Fertility and sterility 2013;100:1695-703.
2. Pandian Z, Gibreel A, Bhattacharya S. In vitro fertilisation for unexplained subfertility. The Cochrane database of systematic reviews 2012:CD003357.
3. Bhattacharya S, Kamath MS. Reducing multiple births in assisted reproduction technology. Best practice & research Clinical obstetrics & gynaecology 2014;28:191-9.
4. Ubaldi FM, Capalbo A, Colamaria S, et al. Reduction of multiple pregnancies in the advanced maternal age population after implementation of an elective single embryo transfer policy coupled with enhanced embryo selection: pre- and post-intervention study. Hum Reprod 2015;30:2097-106.
5. Macklon NS, Geraedts JP, Fauser BC. Conception to ongoing pregnancy: the 'black box' of early pregnancy loss. Human reproduction update 2002;8:333-43.
6. Farfalli VI, Magli MC, Ferraretti AP, Gianaroli L. Role of aneuploidy on embryo implantation. Gynecologic and obstetric investigation 2007;64:161-5.
7. Sugiura-Ogasawara M, Ozaki Y, Katano K, Suzumori N, Kitaori T, Mizutani E. Abnormal embryonic karyotype is the most frequent cause of recurrent miscarriage. Hum Reprod 2012;27:2297-303.
8. Margalioth EJ, Ben-Chetrit A, Gal M, Eldar-Geva T. Investigation and treatment of repeated implantation failure following IVF-ET. Human reproduction 2006;21:3036-43.
9. Forman EJ, Hong KH, Ferry KM, et al. In vitro fertilization with single euploid blastocyst transfer: a randomized controlled trial. Fertility and sterility 2013;100:100-7.e1.
10. Capalbo A, Rienzi L, Cimadomo D, et al. Correlation between standard blastocyst morphology, euploidy and implantation: an observational study in two centers involving 956 screened blastocysts. Hum Reprod 2014;29:1173-81.
11. Demko ZP, Simon AL, McCoy RC, Petrov DA, Rabinowitz M. Effects of maternal age on euploidy rates in a large cohort of embryos analyzed with 24-chromosome single-nucleotide polymorphism-based preimplantation genetic screening. Fertility and sterility 2016;105:1307-13.
12. Franasiak JM, Forman EJ, Hong KH, et al. The nature of aneuploidy with increasing age of the female partner: a review of 15,169 consecutive trophectoderm biopsies evaluated with comprehensive chromosomal screening. Fertility and sterility 2014;101:656-63 e1.
13. Yang Z, Liu J, Collins GS, et al. Selection of single blastocysts for fresh transfer via standard morphology assessment alone and with array CGH for good prognosis IVF patients: results from a randomized pilot study. Molecular cytogenetics 2012;5:24.
14. Schoolcraft WB, Katz-Jaffe MG. Comprehensive chromosome screening of

trophectoderm with vitrification facilitates elective single-embryo transfer for infertile women with advanced maternal age. Fertility and sterility 2013;100:615-9.
15. Wu MY, Chao KH, Chen CD, Chang LJ, Chen SU, Yang YS. Current status of comprehensive chromosome screening for elective single-embryo transfer. Obstetrics and gynecology international 2014;2014:581783.
16. Checa MA, Alonso-Coello P, Sola I, Robles A, Carreras R, Balasch J. IVF/ICSI with or without preimplantation genetic screening for aneuploidy in couples without genetic disorders: a systematic review and meta-analysis. Journal of assisted reproduction and genetics 2009;26:273-83.
17. Meyer LR, Klipstein S, Hazlett WD, Nasta T, Mangan P, Karande VC. A prospective randomized controlled trial of preimplantation genetic screening in the "good prognosis" patient. Fertility and sterility 2009;91:1731-8.
18. Staessen C, Verpoest W, Donoso P, et al. Preimplantation genetic screening does not improve delivery rate in women under the age of 36 following single-embryo transfer. Hum Reprod 2008;23:2818-25.
19. Mastenbroek S, Twisk M, van Echten-Arends J, et al. In vitro fertilization with preimplantation genetic screening. The New England journal of medicine 2007;357:9-17.
20. Mastenbroek S, Twisk M, van der Veen F, Repping S. Preimplantation genetic screening: a systematic review and meta-analysis of RCTs. Human reproduction update 2011;17:454-66.
21. Anderson RA, Pickering S. The current status of preimplantation genetic screening: British Fertility Society Policy and Practice Guidelines. Human fertility (Cambridge, England) 2008;11:71-5.
22. Harton G, Braude P, Lashwood A, et al. ESHRE PGD consortium best practice guidelines for organization of a PGD centre for PGD/preimplantation genetic screening. Hum Reprod 2011;26:14-24.
23. Practice Committee of the Society for Assisted Reproductive Technology and Practice Committee of the American Society for Reproductive Medicine.Preimplantation genetic testing: a Practice Committee opinion. Fertility and sterility 2007;88:1497-504.
24. Geraedts JP. Does additional hybridization also improve preimplantation genetic screening results? Expert review of molecular diagnostics 2010;10:981-5.
25. Schoolcraft WB, Fragouli E, Stevens J, Munne S, Katz-Jaffe MG, Wells D. Clinical application of comprehensive chromosomal screening at the blastocyst stage. Fertility and sterility 2010;94:1700-6.
26. Capalbo A, Ubaldi FM, Cimadomo D, et al. Consistent and reproducible outcomes of blastocyst biopsy and aneuploidy screening across different biopsy practitioners: a multicentre study involving 2586 embryo biopsies. Hum Reprod 2016;31:199-208.
27. Scott RT, Jr., Upham KM, Forman EJ, Zhao T, Treff NR. Cleavage-stage biopsy significantly impairs human embryonic implantation potential while blastocyst biopsy does not: a randomized and paired clinical trial. Fertility and sterility 2013;100:624-30.
28. Fiorentino F, Biricik A, Bono S, et al. Development and validation of a next-generation sequencing-based protocol for 24-chromosome aneuploidy screening of embryos. Fertility and sterility 2014;101:1375-82.

29. Huang J, Yan L, Lu S, Zhao N, Xie XS, Qiao J. Validation of a next-generation sequencing-based protocol for 24-chromosome aneuploidy screening of blastocysts. Fertility and sterility 2016;105:1532-6.
30. Kung A, Munne S, Bankowski B, Coates A, Wells D. Validation of next-generation sequencing for comprehensive chromosome screening of embryos. Reproductive biomedicine online 2015;31:760-9.
31. Tan Y, Yin X, Zhang S, et al. Clinical outcome of preimplantation genetic diagnosis and screening using next generation sequencing. GigaScience 2014;3:30.
32. Yang Z, Lin J, Zhang J, et al. Randomized comparison of next-generation sequencing and array comparative genomic hybridization for preimplantation genetic screening: a pilot study. BMC medical genomics 2015;8:30.
33. Dahdouh EM, Balayla J, Garcia-Velasco JA. Comprehensive chromosome screening improves embryo selection: a meta-analysis. Fertility and sterility 2015;104:1503-12.
34. Chen M, Wei S, Hu J, Quan S. Can Comprehensive Chromosome Screening Technology Improve IVF/ICSI Outcomes? A Meta-Analysis. PloS one 2015;10:e0140779.
35. Lee E, Illingworth P, Wilton L, Chambers GM. The clinical effectiveness of preimplantation genetic diagnosis for aneuploidy in all 24 chromosomes (PGD-A): systematic review. Hum Reprod 2015;30:473-83.
36. Schoolcraft.W.B. SE, Minjarez.D, Gustofson.R.L, Scott Jr., Katz-Jaffe.M.G,. Comprehensive chromosome screening (CCS) with vitrification results in improved clinical outcome in women >35 years: a randomized control trial. Fertility and sterility 2012;98:Supplement, Page S1

37. Forman EJ, Hong KH, Franasiak JM, Scott RT, Jr. Obstetrical and neonatal outcomes from the BEST Trial: single embryo transfer with aneuploidy screening improves outcomes after in vitro fertilization without compromising delivery rates. American journal of obstetrics and gynecology 2014;210:157 e1-6.
38. Scott RT, Jr., Upham KM, Forman EJ, et al. Blastocyst biopsy with comprehensive chromosome screening and fresh embryo transfer significantly increases in vitro fertilization implantation and delivery rates: a randomized controlled trial. Fertility and sterility 2013;100:697-703.
39. Murugappan G, Ohno MS, Lathi RB. Cost-effectiveness analysis of preimplantation genetic screening and in vitro fertilization versus expectant management in patients with unexplained recurrent pregnancy loss. Fertility and sterility 2015;103:1215-20.
40. Mersereau JE, Plunkett BA, Cedars MI. Preimplantation genetic screening in older women: a cost-effectiveness analysis. Fertility and sterility 2008;90:592-8.
41. Greco E, Minasi MG, Fiorentino F. Healthy Babies after Intrauterine Transfer of Mosaic Aneuploid Blastocysts. The New England journal of medicine 2015;373:2089-90.
42. Kang HJ, Melnick AP, Stewart JD, Xu K, Rosenwaks Z. Preimplantation genetic screening: who benefits? Fertility and sterility 2016;106:597-602.
43. Kushnir VA, Darmon SK, Albertini DF, Barad DH, Gleicher N. Effectiveness of in vitro fertilization with preimplantation genetic screening: a reanalysis of United States assisted

reproductive technology data 2011-2012. Fertility and sterility 2016;106:75-9.
44. Gleicher N, Kushnir VA, Barad DH. Preimplantation genetic screening (PGS) still in search of a clinical application: a systematic review. Reproductive biology and endocrinology : RB&E 2014;12:22.
45. Murugappan G, Shahine LK, Perfetto CO, Hickok LR, Lathi RB. Intent to treat analysis of in vitro fertilization and preimplantation genetic screening versus expectant management in patients with recurrent pregnancy loss. Hum Reprod 2016.
46. Shahine LK, Lathi RB. Embryo selection with preimplantation chromosomal screening in patients with recurrent pregnancy loss. Seminars in reproductive medicine 2014;32:93-9.
47. Mastenbroek S, Repping S. Preimplantation genetic screening: back to the future. Hum Reprod 2014;29:1846-50.
48. Orvieto R. Preimplantation genetic screening- the required RCT that has not yet been carried out. Reproductive biology and endocrinology : RB&E 2016;14:35.
49. Templeton A, Morris JK. Reducing the risk of multiple births by transfer of two embryos after in vitro fertilization. The New England journal of medicine 1998;339:573-7.
50. Medicine. PCoSfARTPCoASfR. Elective single-embryo transfer. Fertility and sterility 2012;97:835-42.
51. Vilska S, Tiitinen A, Hyden-Granskog C, Hovatta O. Elective transfer of one embryo results in an acceptable pregnancy rate and eliminates the risk of multiple birth. Human reproduction 1999;14:2392-5.
52. Glujovsky D, Blake D, Farquhar C, Bardach A. Cleavage stage versus blastocyst stage embryo transfer in assisted reproductive technology. The Cochrane database of systematic reviews 2012:Cd002118.
53. Papanikolaou EG, Camus M, Kolibianakis EM, Van Landuyt L, Van Steirteghem A, Devroey P. In vitro fertilization with single blastocyst-stage versus single cleavage-stage embryos. The New England journal of medicine 2006;354:1139-46.
54. Gardner DK, Surrey E, Minjarez D, Leitz A, Stevens J, Schoolcraft WB. Single blastocyst transfer: a prospective randomized trial. Fertility and sterility 2004;81:551-5.
55. Maheshwari A, Griffiths S, Bhattacharya S. Global variations in the uptake of single embryo transfer. Hum Reprod Update 2011;17:107-20.
56. Devroey P, Bourgain C, Macklon NS, Fauser BC. Reproductive biology and IVF: ovarian stimulation and endometrial receptivity. Trends in endocrinology and metabolism: TEM 2004;15:84-90.
57. Choux C, Carmignac V, Bruno C, Sagot P, Vaiman D, Fauque P. The placenta: phenotypic and epigenetic modifications induced by Assisted Reproductive Technologies throughout pregnancy. Clinical epigenetics 2015;7:87.
58. Maheshwari A, Pandey S, Shetty A, Hamilton M, Bhattacharya S. Obstetric and perinatal outcomes in singleton pregnancies resulting from the transfer of frozen thawed versus fresh embryos generated through in vitro fertilization treatment: a systematic review and meta-analysis. Fertility and sterility 2012;98:368-77 e1-9.
59. Chen Z-J, Shi Y, Sun Y, et al. Fresh versus Frozen Embryos for Infertility in the Polycystic Ovary Syndrome. The New England journal of medicine 2016;375:523-33.

60. Gardner DK, Schoolcraft WB. In vitro culture of human blastocyst. In: JR M, ed. Toward Reproductive Certainty: Infertility and Genetics Beyond 1999. Carnforth, UK: Parthenon Press; 1999:378–88.
61. Gardner DK, Lane M, Stevens J, Schlenker T, Schoolcraft WB. Blastocyst score affects implantation and pregnancy outcome: towards a single blastocyst transfer. Fertility and sterility 2000;73:1155-8.
62. Minasi MG, Colasante A, Riccio T, et al. Correlation between aneuploidy, standard morphology evaluation and morphokinetic development in 1730 biopsied blastocysts: a consecutive case series study. Hum Reprod 2016;31:2245-54.
63. Wirleitner B, Schuff M, Stecher A, Murtinger M, Vanderzwalmen P. Pregnancy and birth outcomes following fresh or vitrified embryo transfer according to blastocyst morphology and expansion stage, and culturing strategy for delayed development. Hum Reprod 2016;31:1685-95.
64. van Echten-Arends J, Mastenbroek S, Sikkema-Raddatz B, et al. Chromosomal mosaicism in human preimplantation embryos: a systematic review. Human reproduction update 2011;17:620-7.
65. Hook EB. Prevalence of chromosome abnormalities during human gestation and implications for studies of environmental mutagens. Lancet (London, England) 1981;2:169-72.
66. Bolton H, Graham SJ, Van der Aa N, et al. Mouse model of chromosome mosaicism reveals lineage-specific depletion of aneuploid cells and normal developmental potential. Nature communications 2016;7:11165.
67. Chen X, Zhang J, Wu X, et al. Trophectoderm morphology predicts outcomes of pregnancy in vitrified-warmed single-blastocyst transfer cycle in a Chinese population. Journal of assisted reproduction and genetics 2014;31:1475-81.
68. Schieve LA, Meikle SF, Peterson HB, Jeng G, Burnett NM, Wilcox LS. Does assisted hatching pose a risk for monozygotic twinning in pregnancies conceived through in vitro fertilization? Fertility and sterility 2000;74:288-94.

Supplemental Appendix

Table 1. Comparison among different methods for PGS

Techniques	Brief overview	Strengths	Drawbacks	Current status in clinic
FISH	Fluorescence in situ hybridization is a cytogenetic technique that uses fluorescent probes that bind to only those parts of the chromosome with a high degree of sequence complementarity.	1. FISH can be used in chromosome analysis of embryos produced by Robertsonian translocation carriers.<br>2. Chromosome-specific centromeric and telomeric probes can be designed to distinguish balanced from unbalanced embryos produced by translocation carriers.<br>3. It is cheap and time saving.	1. Difficulties in obtaining the required probes with necessary fluorochrome/reporter molecule labeling, split signals/signal overlap, and probe target polymorphisms.<br>2. Limited by the number of fluorescently labelled probes, only aneuploidy for chromosome 13, 15–18, 21, 22, X and Y could be detected. But these chromosomes need to be assessed over multiple rounds of hybridization.<br>3. Poorly predictive of aneuploidy as a result of a high rate of false positives when used on single cells<br>4. Influenced by many factors such as the operator's skill in cell-spreading, the hybridization efficiency and the probe quality.<br>5. Blastomere biopsy at Day 3 cleavage stage embryos does impair the implantation potential.	1. It has been replaced by more reliable technologies
aCGH	Comparative genomic hybridization microarrays involve	1. It has a better resolution than qPCR.<br>2. It can examine all 24	1. Time consuming and embryos need to be frozen pending the results of the CGH, with associated compro-	1. Now there are a lot of aCGH platforms available in clinic.

	differential labeling and mixing of biopsy DNA with control DNA prior to hybridization and interpretation of ratios of red and green (two-color) fluorescence upon completion	chromosomes simultaneously.	mise of embryo quality.<br>2. Expensive.<br>3. Rely on the unknown database, lower coverage than NGS.<br>4. Lower sequencing depth than NGS.<br>5. It is not possible to use aCGH for balanced translocation, haploid or polyploid embryo, low-grade mosaicism and single mutations	
SNP microarray	Single nucleotide polymorphism microarrays involve hybridization of only biopsy DNA (single color) followed by computational comparison of signal intensities to those obtained on separate control DNA hybridized arrays.	1. Time saving for fresh embryo transfer<br>2. SNP arrays have the added advantage to identify the parental alleles in the embryo as to exclude uniparental disomy (UPD).	1. Expensive.	1. SNP array is commonly used as a gold standard.<br>2. It can provide genotypic information about single gene disorders, UPD, loss of heterozygosity (LOH), DNA fingerprinting and determination of the parental and cell division origins of aneuploidy.
qPCR	Quantitative real-time PCR	1. It does not require whole genome amplification.<br>2. Much more time saving method from which results are available within 4 h of the biopsy allowing fresh blastocyst	Not reported.	1. It has been used for detection of aneuploidy (e.g., trisomy 21) of fetal cells in maternal serum.

		transfer after trophectoderm biopsy.		
NGS	After the whole genome amplification, the material is cut into small DNA fragments (of 100–200 base pairs) and placed on a 2 × 2 cm ‘chip’. A sequence of each fragment is then compared to a reference sequence. The results are prepared by a computer.	1. High coverage rate.<br>2. High sequencing depth.<br>3. NGS can analyze aneuploidy or translocation of all chromosomes and also mutations responsible for any single-gene disease.<br>4. High throughput by parallel analysis of multiple samples in a single sequencing run<br>5. Reduced cost.	1. The process is complex.<br>2. Time-consuming<br>3. A lot of de novo mutation will be found, so it needs further consideration in explaining the result to the patient.<br>4. The analysis of the output data needs to base on the known database.	Currently, NGS is thought to be a future and target technology for PGD/PGS.

Table 3. List of registered randomized clinical trial regrading PGS

PI	Clinical trial registration number and link	Status	Treatment groups	Main patient population	Sample Size	Primary Outcome	Secondary outcomes	Live Birth rates	Cumulative live birth rates
Reprogenetics Santiago Munne, Ph.D	NCT01946945	Unknown	Experimental: PGS-Frozen SET All embryos will be hatched on day 3. Patients will have hatching blastocysts biopsied on day 5/6. Embryos will be vitrified. Patients will have a single hatching euploid blastocyst replaced on a thawed cycle. Control - Standard ART treat-	Age of 18-42 Inclusion Criteria: 1. All patients medically cleared to do a fresh or frozen embryo transfer. 2. Age up to 42 years Exclusion Criteria: 1. Microsurgical epididymal sperm aspiration (MESA) and Testicular sperm extraction (TESE) patients 2. At least one partner carrier of a chromosomal or genetic	240	Improvement in ongoing implantation rates	Determine specificity and sensitivity rates Correlation of Mitochondrial DNA and implantation	No	No

PI	Clinical trial registration number and link	Status	Treatment groups	Main patient population	Sample Size	Primary Outcome	Secondary outcomes	Live Birth rates	Cumulative live birth rates
			ment	disease<br>3. Abnormal ovarian reserve, defined as follicle stimulating hormone (FSH) of >10 IU/L on day 2-4 of the cycle and anti-mullerian hormone (AMH) < 1ng /ml (If only one of the two parameters altered then patients is acceptable). This is based on Mandy Katz abstract at American Society for Reproductive Medicine (ASRM) 2011 where they showed					

PI	Clinical trial registration number and link	Status	Treatment groups	Main patient population	Sample Size	Primary Outcome	Secondary outcomes	Live Birth rates	Cumulative live birth rates
				that these patients have 35% chance of having no euploid embryos - They are excluded only to make the study size smaller, otherwise, if an euploid embryo is found in these patients, they implant as well as patients with normal ovarian reserve. Not all centers do AMH testing - we recommend first to run FSH and only test AMH if FSH is abnormal.<br>4. Egg donor cycle (sperm do-					

PI	Clinical trial registration number and link	Status	Treatment groups	Main patient population	Sample Size	Primary Outcome	Secondary outcomes	Live Birth rates	Cumulative live birth rates
				nor is acceptable)<br>5. Gender selection cycles<br>6. Thaw cycles					
Reproductive Medicine Associates of New Jersey Richard T Scott, M.D., HCLD	NCT02032264	Active, not recruiting	Experimental: NGS based CCS-SET or DET Trophectoderm biopsy will be performed on all blastocysts and CCS via next generation sequencing screening performed on biopsy samples. Patients will proceed with a single or double embryo	Normal ovarian reserve(18-42) Inclusion Criteria:<br>1. Patient undergoing IVF/CCS (no PGD banking)<br>2. Patient meets ASRM guidelines for Double Embryo Transfer (DET)<br>3. Donor Sperm OK<br>4. AMH ≥ 1.2<br>5. FSH ≤ 12	250	Impact of next generation sequencing on the embryos produced from IVF on implantation rates	Delivery Rates	Yes（2nd）	No

PI	Clinical trial registration number and link	Status	Treatment groups	Main patient population	Sample Size	Primary Outcome	Secondary outcomes	Live Birth rates	Cumulative live birth rates
			transfer of the one or two morphologically best euploid embryos<br>Control: SET or DET<br>The patients in this group will proceed with a single or double embryo transfer of the one or two morphologically best embryos.	6. BAFC ⩾12<br>7. Max 1 prior failed IVF cycle for patients 35-45 years old<br>8. Patient <35 years old MUST have 1 prior failed IVF cycle<br>Exclusion Criteria:<br>1. Chronic endometrial insufficiency<br>2. Use of oocyte donor or gestational carriers<br>3. Medical contraindications to Double Embryo Transfer (DET)					

PI	Clinical trial registration number and link	Status	Treatment groups	Main patient population	Sample Size	Primary Outcome	Secondary outcomes	Live Birth rates	Cumulative live birth rates
				4. Male Factor (<100,000 sperm or surgical sperm)<br>5. Communicating hydrosalpinx (on HSG)<br>6. Single gene disorders or sex selection					
Illumina, Inc<br>Amy Mueller, MD	NCT02268786	Active, not recruiting	Experimental:NGS based PGS-SET<br>Intent to transfer single euploid embryo based on NGS testing (VeriSeq™ PGS) of biopsied blastocysts<br>Control:No	Normal ovarian reserve(25-40)<br>Inclusion Criteria:<br>1. Patient undergoing IVF<br>2. At least 2 blastocysts suitable for biopsy on day 5 or 6 of embryo development<br>Exclusion Criteria:	600	Ongoing Pregnancy(20w)	Fetal Aneuploidy Status	No	No

PI	Clinical trial registration number and link	Status	Treatment groups	Main patient population	Sample Size	Primary Outcome	Secondary outcomes	Live Birth rates	Cumulative live birth rates
			PGS-SET Intent to transfer single embryo based on morphological assessment according to the Gardner scoring system (noPGS)	1. History of more than two prior implantation failure following IVF<br>2. History of more than one miscarriage of viable pregnancy<br>3. One or both partners known to be carrier(s) of a chromosomal abnormality<br>4. Known genetic carrier couple and/or one or both partners carrier of a known autosomal dominant disorder					

PI	Clinical trial registration number and link	Status	Treatment groups	Main patient population	Sample Size	Primary Outcome	Secondary outcomes	Live Birth rates	Cumulative live birth rates
				5. Any other non-study related preimplantation genetic testing<br>6. Use of donor oocytes<br>7. Use of gestational carrier (surrogate or donor egg recipient).<br>8. Severe oligospermia (<1,000,000 sperm/ml); Surgical Sperm Retrieval for reasons other than post-vasectomy and CAVD<br>9. Low ovarian reserve with (FSH) >10 IU/L on day 2-4 of a					

PI	Clinical trial registration number and link	Status	Treatment groups	Main patient population	Sample Size	Primary Outcome	Secondary outcomes	Live Birth rates	Cumulative live birth rates
				prior menstrual cycle and/or (AMH) <7 pmol/L (or <1 ng/ml)<br>10. Gender selection cycles<br>11. Concurrent participation in another clinical trial					
Reproductive Medicine Associates of New Jersey Richard T Scott, MD	NCT01219283	Completed （Scott ）	Experimental: PGD-DET Receive PGD in addition to their planned IVF Cycle. 2 morphologically best PGD normal embryos are transferred. Control: no PGD-DET	Normal ovarian reserve(21-42), ≤ 1 previous IVF failure<br>Inclusion Criteria:<br>1. Age of female partner of < 43 years<br>2. Normal day-three FSH level (< 15	334	Sustained Implantation Rate Per Embryo Transferred	Clinical Pregnancy Rate and Delivery Rate per Treatment Cycle	Yes(2nd) 84.7% vs 67.5%	No

PI	Clinical trial registration number and link	Status	Treatment groups	Main patient population	Sample Size	Primary Outcome	Secondary outcomes	Live Birth rates	Cumulative live birth rates
			Receive their planned IVF treatment without PGD. 2 morphologically best embryos are transferred.	mIU/mL)<br>3. Normal uterine cavity<br>4. Sufficient ejaculated spermatozoa in male partner for ART<br>5. Maximum of one prior failed IVF cycle<br>Exclusion Criteria:<br>1. FSH level ⩾ 15 mIU/mL<br>2. BMI greater than 32 kg/m2<br>3. Contraindication to gonadotropin stimulation<br>4. Unevaluated					

PI	Clinical trial registration number and link	Status	Treatment groups	Main patient population	Sample Size	Primary Outcome	Secondary outcomes	Live Birth rates	Cumulative live birth rates
				Ovarian mass<br>5. Need for surgical sperm removal<br>6. Any contraindication to undergoing in vitro fertilization<br>7. Age greater than 43 years<br>8. Presence of hydrosalpinges which communicate with the endometrial cavity<br>9. Clinical indication for PGD (undergoing IVF with PGD to rule out a known genetic					

PI	Clinical trial registration number and link	Status	Treatment groups	Main patient population	Sample Size	Primary Outcome	Secondary outcomes	Live Birth rates	Cumulative live birth rates
				defect)					
Ferring Pharmaceuticals<br><br>Richard T Scott, MD	NCT01408433	Completed（Forman）	Experimental: qPCR-based CCS and SET<br>Control: Morphological assessment and DET	Normal ovarian reserve(age ＜42)<br>Inclusion Criteria:<br>1. ≤ 1 previous IVF failure<br>2. Maximum prior day 3 follicle stimulation hormone (FSH) level of 12;<br>Minimum antimullerian hormone (AMH)of 1.2 within 1 year<br>3. Normal uterine cavity demonstrated by saline sonogram, hysterosalpingogram or hysteroscopy	89 vs.86	Ongoing PR (>24 wk),	Twin live birth rate<br>Sustained implantation rate<br>Live birth rate per randomized patient<br>Live birth rate per embryo transfer	Yes(follow-up) 61% vs 65%	Yes(follow-up of through the fresh cycle and up to 1 frozen transfer)<br>69% vs 72%

PI	Clinical trial registration number and link	Status	Treatment groups	Main patient population	Sample Size	Primary Outcome	Secondary outcomes	Live Birth rates	Cumulative live birth rates
				within 1 year.<br>4. Male partner with greater than 100,000 total motile spermatozoa. Donor sperm ok.<br>5. Body Mass Index (BMI) less than or equal to 30 kg/m2.<br>Exclusion Criteria:<br>1. Diagnosis of chronic anovulation (cycles typically longer than 90 days)<br>2. Diagnosis of endometrial insufficiency- prior cycle					

PI	Clinical trial registration number and link	Status	Treatment groups	Main patient population	Sample Size	Primary Outcome	Secondary outcomes	Live Birth rates	Cumulative live birth rates
				with endometrial thickness less than 6mm, abnormal endometrial echotexture, persistent endometrial fluid.<br>3. Clinical indication of aneuploidy screening (i.e. history of loss of chromosomally abnormal pregnancies)<br>4. Clinical indication for PGD for single-gene disorder (i.e. PGD is needed to select					

PI	Clinical trial registration number and link	Status	Treatment groups	Main patient population	Sample Size	Primary Outcome	Secondary outcomes	Live Birth rates	Cumulative live birth rates
				against the transfer of embryos affected with a specific condition)<br>5. Use of testicular aspiration or biopsy procedures to obtain sperm<br>6. Unevaluated ovarian mass or surgically confirmed stage IV endometriosis<br>7. Presence of hydrosalpinges which communicate with the endometrial cavity<br>8. Any contrain-					

PI	Clinical trial registration number and link	Status	Treatment groups	Main patient population	Sample Size	Primary Outcome	Secondary outcomes	Live Birth rates	Cumulative live birth rates
				dication to undergoing in vitro fertilization					
Reprogenetics Santiago Munne, PhD	NCT01332643	Terminated	Experimental: CGH based PGS-,frozen SET The test group will consist of patients undergoing blastocyst biopsy followed by vitrification (embryo freezing), and in which the biopsied cells will be analyzed with a comprehensive chromosome	Normal ovarian reserve(30-42)<br>Inclusion Criteria:<br>1. Couples with women 30-42 years of age<br>2. Follicle Stimulating Hormone (FSH) level <11IU/L on day 3 of cycle.<br>Exclusion Criteria<br>1. TESA and TESE patients<br>2. Couples' carriers of chromosomal or genetic diseases<br>3. Couples that	240	Implantation rate	Miscarriage rate<br>Pregnancy rate per transfer<br>Pregnancy rate per retrieval<br>live birth rate	Yes(2nd)	No

PI	Clinical trial registration number and link	Status	Treatment groups	Main patient population	Sample Size	Primary Outcome	Secondary outcomes	Live Birth rates	Cumulative live birth rates
			analysis technique (array Comparative Genome hybridization or aCGH) and only one chromosomally normal embryo will be replaced in a thawed cycle. Control:SET The control group will consist of patients in which one embryo will be replaced on day 5 based on morphological and developmental	produce less than eight antral follicles on day 2-4 of cycle<br>4. Patients will be excluded if they produce no blastocysts by day 5					

PI	Clinical trial registration number and link	Status	Treatment groups	Main patient population	Sample Size	Primary Outcome	Secondary outcomes	Live Birth rates	Cumulative live birth rates
			characteristics, and the other embryos reaching blastocyst stage will be vitrified.						

PI	Clinical trial registration number and link	Status	Treatment groups	Main patient population	Sample Size	Primary Outcome	Secondary outcomes	Live Birth rates	Cumulative live birth rates
Reprogenetics Santiago Munne, PhD	NCT01546350	Suspended	Experimental: aCGH based PGD-SET Patients will have grade A,B or C blastocysts hatched on day 5, biopsied on day 5, analyzed by array CGH, and a single euploid embryo transferred on day 6. Control: regular ART treatment-DET Patients will have up to two embryos replaced on day 5 based on morphological and developmental characteristics, and the	Normal ovarian reserve (Age of 33-42)<br>Inclusion Criteria:<br>1. Maternal age 33 to 42 years old (included)<br>Exclusion Criteria:<br>1. MESA and TESE patients<br>2. At least one partner carrier of a chromosomal or genetic disease<br>3. Abnormal ovarian reserve, defined as FSH of >10 IU/L on day 2-4 of the cycle and AMH < 1ng /ml (If only one of the two parameters altered then patients is acceptable).<br>4. Egg donor cycle (sperm do-	200	Implantation rate	Spontaneous miscarriage rate ongoing pregnancy rate Multiple pregnancy rate	No	No

PI	Clinical trial registration number and link	Status	Treatment groups	Main patient population	Sample Size	Primary Outcome	Secondary outcomes	Live Birth rates	Cumulative live birth rates
Igenomix Carlos Simon	NCT01571076	unkown	PGS-CGH Experimental: PGS PGS of day three biopsies and consequent embryo transfer on on Day 5 (blastocyst) Control: No PGS Prolonged culture, no PGS, for Day 5 (blastocyst) embryo transfer for the Advanced Age group	AMA(38-41) Inclusion Criteria: ADVANCED AGE and SEVERE MALE FACTOR:<br>1. Women's Age: 38- 41 years old (both included)<br>2. Men's Age: 18-60 years old (both included)<br>3. Ovulation triggered with human chorionic gonadotrophin (hCG) (Ovitrelle, Merck-Serono, Madrid)<br>4. Number of Oocytes metaphase II (MII): $\geqslant 5$ (fresh, not vitrified)	240	Ongoing Implantation and Pregnancy Rate	No	No	No

PI	Clinical trial registration number and link	Status	Treatment groups	Main patient population	Sample Size	Primary Outcome	Secondary outcomes	Live Birth rates	Cumulative live birth rates
				5. Quality of semen: ⩾ 5 millions spermatozoids/ml<br><br>Exclusion Criteria:<br>1. Number of Oocytes metaphase II (MII): <5 oocytes<br>2. Number of Oocytes obtained: >20<br>3. Estradiol on the day of human chorionic gonadotrophin (hCG) >3000 pgr/ml administration.<br>4. Progesterone on the day of hCG >1,5 pmol/l admin-					

PI	Clinical trial registration number and link	Status	Treatment groups	Main patient population	Sample Size	Primary Outcome	Secondary outcomes	Live Birth rates	Cumulative live birth rates
				istration<br>5. ⩾ 2 previous miscarriages: (biochemical,clinical,ectopic or a combination)<br>6. Any uncorrected alteration in a previous study.<br>7. Other indications of (Preimplantation Genetic Diagnosis.Screening) PGD-S, such as monogenic illnesses, translocations, repeated implantation failure, repeated miscarriages, etc.					

PI	Clinical trial registration number and link	Status	Treatment groups	Main patient population	Sample Size	Primary Outcome	Secondary outcomes	Live Birth rates	Cumulative live birth rates
Reproductive & Genetic Hospital of CITIC-Xiangya	NCT02868528	Not recruiting	PGS-NGS vs.IVF Experimental: PGS group After blastocyst culture, blastocyst embryo trophoblast biopsy will be performed and chromosome screening with NGS technology, at the same time, the blastocysts will be frozen, then the blastocysts with normal chromosome will be thawed and	AMA(37-44) with normal ovarian reserve;AFC⩾10;AMH⩾2.0<br>Inclusion Criteria:<br>1. Premenopausal females, age ⩾ 37 years ⩽44 years<br>2. Have given birth to a healthy baby<br>3. Bilateral ovaries<br>4. Antral follicle count(AFC)⩾10,and Anti Mullerian Hormone (AMH)⩾2.0 ng/ml<br>Exclusion Criteria:<br>1. Endometriosis disease	238	live birth rate	cancellation rate Pregnancy Rate abortion rate	Yes	No

PI	Clinical trial registration number and link	Status	Treatment groups	Main patient population	Sample Size	Primary Outcome	Secondary outcomes	Live Birth rates	Cumulative live birth rates
			transferred. Control: After blastocyst culture, blastocysts will be transferred	2. Intrauterine adhesions history; intrauterine membrane polyp, tuberculosis and inflammation,<br>3. Uterine malformation, multiple uterine myoma, uterine intramural myoma >3cm, submucous myoma;<br>4. Unprocessed hydrosalpinx<br>5. Adverse reproductive history; greater than or equal to 2 times history of unexplained abortion					

PI	Clinical trial registration number and link	Status	Treatment groups	Main patient population	Sample Size	Primary Outcome	Secondary outcomes	Live Birth rates	Cumulative live birth rates
				6. Chromosomal abnormalities or other genetic disease<br>7. Infertility caused by male factors, such as puncture testicular, SRT did not see the class A and class B sperm<br>8. Without high quality embryos in past controlled ovarian hyperstimulation (COH)cycles.<br>9. Patients with poor ovarian response, the standard of poor ovarian response accords					

PI	Clinical trial registration number and link	Status	Treatment groups	Main patient population	Sample Size	Primary Outcome	Secondary outcomes	Live Birth rates	Cumulative live birth rates
				with Bolognacriteria standard, that is at least meet 2 among the following 3:<br><br>*Elder years (⩾40 years) or have other known inherited or acquired risk factors that may reduce follicle.<br>*History of cancellation of the cycles because of less than 3 follicular development, or history of egg number less than 4 after at least using Follicle-Stimulating Hormone(FSH)					

PI	Clinical trial registration number and link	Status	Treatment groups	Main patient population	Sample Size	Primary Outcome	Secondary outcomes	Live Birth rates	Cumulative live birth rates
				150IU once a day.<br>*Ovarian reserve function test abnormalities, including sinus follicle number less than 5-7 AFC or AMH less than 0.5 to 1.1 ng/ml					
Chinese PLA General Hospital	NCT02353364	recruiting	PGS-NGS Experimental: NGS based on PGS-SET or DET Transfer of 1 or 2 biopsied euploid embryo of high morphological grade based on NGS testing using CNV-	AMA(⩾35)<br>Inclusion Criteria:<br>1. Patient undergoing in vitro fertilization (IVF)<br>2. Normal uterine function by ultrasound and absence of hysteromyoma<br>3. Regular menstrual cycle of	1000	Ongoing pregnancy	Genetic health of the fetus	No	No

PI	Clinical trial registration number and link	Status	Treatment groups	Main patient population	Sample Size	Primary Outcome	Secondary outcomes	Live Birth rates	Cumulative live birth rates
			Seq (PGS)<br>Control: SET or DET Transfer of 1 or 2 non-biopsied embryo of high morphological grade (no PGS)	25-35 days<br>4. Normal hormone levels (WHO standard) for LH, PRL, E2, PROG, TEST and TSH<br>5. FSH 1-12 IU/L and follicle number > 5 on day 2-3 of menstrual cycle<br>6. Minimum of 3 blastocysts on day 5 of embryo development<br>7. Signed consent form<br>Exclusion Criteria:<br>1. Known endometriosis					

PI	Clinical trial registration number and link	Status	Treatment groups	Main patient population	Sample Size	Primary Outcome	Secondary outcomes	Live Birth rates	Cumulative live birth rates
				2. Abnormal vaginal bleeding with no known cause<br>3. Known genital organ system malformation, unsuitable to conceive<br>4. Known currently active pelvic inflammation<br>5. Abnormal liver, kidney lab results, with clinical implications.<br>6. Known endocrine or metabolic disorders (pituitary gland, adrenal glands, pancreas, liver					

PI	Clinical trial registration number and link	Status	Treatment groups	Main patient population	Sample Size	Primary Outcome	Secondary outcomes	Live Birth rates	Cumulative live birth rates
				or kidney）<br>7. Known ovarian, breast, uterine, adrenal glands, pituitary gland or hypothalamus tumor<br>8. Known abnormal cervical cancer lesions, with clinical implications, within one year before PGS<br>9. History of chemo- or radio-therapy<br>10. Seropositive for HIV, Hep B, Hep C or TPPA/RPR (Syphilis)<br>11. Known ovarian					

PI	Clinical trial registration number and link	Status	Treatment groups	Main patient population	Sample Size	Primary Outcome	Secondary outcomes	Live Birth rates	Cumulative live birth rates
				poor response in previous cycles, i.e. after administration of GnRH for > 20 days<br>12. More than 2 implantation failures<br>13. More than 2 miscarriages<br>14. Known altered parental karyotype such as Robertsonian or reciprocal translocation<br>15. Use of sperm or oocyte donors<br>16. Severe male factor (surgical retrieval of sperm)					

PI	Clinical trial registration number and link	Status	Treatment groups	Main patient population	Sample Size	Primary Outcome	Secondary outcomes	Live Birth rates	Cumulative live birth rates
				17. Preimplantation genetic diagnosis cycles for single gene diseases or sex selection<br>18. Participation in other IVF research studies<br>19. Patient refusal or inability to follow the protocol for any good reason, including clinical visit or lab test					
Natera, Inc.	NCT01194531	Terminated	Experimental: PGS on Day 3<br>Control: no PGS testing	AMA(35-42)<br><br>Inclusion Criteria:<br>1. Must agree to a follow-up visit at approximately 4-8 weeks	59	Implantation Rate	No	No	No

PI	Clinical trial registration number and link	Status	Treatment groups	Main patient population	Sample Size	Primary Outcome	Secondary outcomes	Live Birth rates	Cumulative live birth rates
				gestation and a follow up phone call at 20 and 40 weeks gestation/livebirth. For patients assigned to the TEST group must agree to PGD testing and collection of buccal swab sample on newborn<br>2. At least 10 eggs retrieved<br>3. Must agree to use only Ferring products during stimulation<br>4. Normal uterine cavity detected on hysterosal-					

PI	Clinical trial registration number and link	Status	Treatment groups	Main patient population	Sample Size	Primary Outcome	Secondary outcomes	Live Birth rates	Cumulative live birth rates
				pingogram (HSG), saline infusion sonogram (SIS), or hysteroscopy<br>5. Signed consent form<br>Exclusion Criteria:<br>1. FSH ≥10 IU/L within past year prior to screening<br>2. 2 or more previously failed IVF cycles<br>3. Gestational or surrogate carrier, donor oocyte, donor sperm<br>4. History of recurrent preg-					

PI	Clinical trial registration number and link	Status	Treatment groups	Main patient population	Sample Size	Primary Outcome	Secondary outcomes	Live Birth rates	Cumulative live birth rates
				nancy loss (3 or more consecutive miscarriages)<br><br>5. Severe male factor infertility defined as ejaculate sperm of < 1million sperm/ml, or sperm obtained through testicular biopsy<br><br>6. Gender selection as primary indication<br><br>7. Maternal disease that is not clinically stable and known to impact the ability to become pregnant or carry a pregnancy to term (lupus,					

PI	Clinical trial registration number and link	Status	Treatment groups	Main patient population	Sample Size	Primary Outcome	Secondary outcomes	Live Birth rates	Cumulative live birth rates
				chronic liver or kidney disease, body mass index (BMI) >35, uncontrolled hypertension, anti-phospholipid antibody, thrombophilia, insulin dependent diabetes)<br>8. Refusal or inability to comply with the requirements of the Protocol for any reason, including scheduled clinic visits and laboratory tests<br>9. Participation in any experimental drug					

PI	Clinical trial registration number and link	Status	Treatment groups	Main patient population	Sample Size	Primary Outcome	Secondary outcomes	Live Birth rates	Cumulative live birth rates
				study within 30 days prior to Screening<br>10. Prior hypersensitivity to any of the protocol drugs<br>11. Known history conveying increased risk for chromosome abnormality (beyond maternal age) or genetic disease in offspring					
Reproductive Medicine Associates of New Jersey Richard T. Scott, MD,	NCT01977144	recruiting	Experimental:CCS-SET or DET Embryo Transfer with CCS Patients will have either a	Low responder Inclusion Criteria:<br>1. Age of female partner < 43 y/o AMH < 1.1 OR BAFC < 8 (within previous year) Male	400	Delivery rate	pregnancy rate per ET	Yes	No

PI	Clinical trial registration number and link	Status	Treatment groups	Main patient population	Sample Size	Primary Outcome	Secondary outcomes	Live Birth rates	Cumulative live birth rates
HCLD			single or double embryo transfer with CCS tested embryos Control: SET or DET Embryo Transfer without CCS Patients in this group will not have CCS performed on their embryo(s)	must have >100,000 motile sperm BMI < 32<br>Exclusion Criteria:<br>1. Diagnosis of endometrial insufficiency Use of oocyte donor/gestational carriers Use of surgical sperm or DNA Banking Communicating hydosalpinges (HSG) Single gene disorders or sex selection Participation in another study					
ShangHai Ji Ai Genetics & IVF Insti-	NCT02223221	Recruiting	Experimental: SNP based PGS-SET or DET	RSA(18-48)<br>Inclusion Criteria:<br>1. regular menstrual cycles	240	Ongoing pregnancy(12weeks)	Implantation of transferred	Yes(2nd)	Yes(Sec)?

PI	Clinical trial registration number and link	Status	Treatment groups	Main patient population	Sample Size	Primary Outcome	Secondary outcomes	Live Birth rates	Cumulative live birth rates
tute<br>YILUN SUI, MD			IVF/ICSI cycles with PGS. Select embryos by SNP-array based PGS for the number of all chromosomes on day 5, only euploid embryos will be transferred. A maximum of 2 embryos will be transferred for each treatment cycle. Up to 3 treatment cycles will be offered. Control:No PGS- SET or DET	and normal level of E2, P, FSH, LH, T, RPL in the early follicular phase;<br>2. no history of hormone medicine application in the last 3 months;<br>3. no history of poison contact;<br>4. normal uterine and adnexal ultrasonography;<br>5. TORCH(-), chlamydia(-), mycoplasma(-), normal leucorrhoea routine, anti-phospholipid antibody (-), an-			embryo<br>Clinical pregnancy<br>Time to pregnancy<br>Pregnancy outcome including abortion, live birth, multiple births, birth defect, preterm delivery, small-for-gestational age, still birth, maternal compli-		

PI	Clinical trial registration number and link	Status	Treatment groups	Main patient population	Sam ple Size	Primary Outcome	Secondary outcomes	Live Birth rates	Cumulative live birth rates
			IVF/ICSI cycles without PGS. Selection of embryos are based on blastocyst morphology criteria on day 5.<br>A maximum of 2 embryos will be transferred for each treatment cycle. Up to 3 treatment cycles will be offered.	tinuclear antibody(-);<br>6. for the couple, no blood type incompatibility or ABO antibody IgG≤1：64 and normal blood chromosome analysis.<br>Exclusion Criteria:<br>1. hydrosalpinx without operation; endometriosis; polycystic ovary syndrome; adenomyosis; uterine leiomyomata(submucous myoma or non-submucous myoma which size was exceed 4cm and/or			cations		

PI	Clinical trial registration number and link	Status	Treatment groups	Main patient population	Sample Size	Primary Outcome	Secondary outcomes	Live Birth rates	Cumulative live birth rates
				with the compressed endometrium);uterine cavity lesions(such as uterine malformation, intrauterine adhesions, the septate uterus, endometritis etc);<br>2. the former abortion is because of luteal phase defect without treatment;<br>3. thyroid dysfunction or increased CA125 level;<br>4. acute inflammation of genitourinary system or					

PI	Clinical trial registration number and link	Status	Treatment groups	Main patient population	Sample Size	Primary Outcome	Secondary outcomes	Live Birth rates	Cumulative live birth rates
				STD carriers;<br>5. unable to comply with the study procedures.					

CONSENT FOR RESEARCH

Reproductive Medical center, Shandong University

Title of Project: Preimplantation genetic screening versus Conventional In-vitro fertilization for Live birth: A pragmatic randomized controlled clinical trial (PCIL)

You are cordially invited to participate in the above named research study. You need to decide whether you want to participate in or not. Please take your time to make up your mind. Carefully read the following and feel free to ask the investigator any question which you may have.

Why is this study being done?

You are invited to take part in this research because you are going to undergo in-vitro fertilization procedure and are predicted to have a good chance to success.

In vitro fertilization (IVF) is a well-established technology for infertility treatment. Multiple pregnancies are one of main complications of IVF, which are associated with a high risk of maternal and neonatal complications. Single embryo transfer (SET) is the most effective approach to reduce the risk of multiple pregnancies. And at present, SET is recommended for women with good prognosis. The selection of embryo for transfer is crucial to maintain the chance of success. In addition to conventional morphological score, the genetic of embryo is suggested to be an important factor affecting the development potential of embryos. Therefore, preimplantation genetic screening (PGS) is proposed to pick up euploid embryo for transfer. However, PGS is concerned about the inefficiency in embryo utility that may result from discard of embryos with developmental capability. There was no sufficient evidence to prove the efficacy and safety of PGS in terms of cumulative live birth rate after one time of oocyte retrieval.

The purpose of this study is to compare cumulative live birth rate of in-vitro fertilization (IVF) with preimplantation genetic screening (PGS) versus IVF alone.

Who should be in this study?
You will be included in this study if you have the following:

- You are undergoing your first cycle of IVF or ICSI;
- You age 20 to 37 years;

You will not be included in this study if you have the following:

- Uterine cavity abnormality, such as uterine malformation (uterus unicornis, bicornis, or duplex); untreated uterus septus, adenomyosis, submucous myoma, or multiple endometrial polyps; or with history of intrauterine adhesions.
- Plan to undergo PGD because of parental abnormal karyotype or diagnosed with monogenic disease;
- Plan to use donated oocytes or sperm to achieve pregnancy;
- Medical condition that represent contraindication to assisted reproductive technology and/or pregnancy;

According to the study protocol, if you don't obtain sufficient oocytes or embryos, you will be taken out of in this study. The subsequent treatment for will be performed according to the present clinical routine.

What will I be asked to do?

To participate in this study, you will undergo are standard protocol for ovarian stimulation. Fertilization is achieved by intracytoplasmic sperm injection. All embryos will be frozen in the fresh cycle and subsequent frozen embryo transfer will be performed. And single frozen embryo will be transferred each time for the first 3 transfer cycles. The research part is that you will be randomized to PGS or IVF group on day 5 of embryo culture. Subjects in the PGS group will have 3 blastocysts biopsied and sequenced, euploid embryos will be subsequently transferred one by one. Subjects in the IVF group will have 3 blastocysts selected according to morphologic score for the first 3 transfers. If you achieve clinical pregnancy, follow-ups with a little more frequency than routine will be required with the purpose of acquiring pregnancy complications information.

How long will I be in the study?

The treatments will last for 3 month. If you obtain pregnancy after the first transfer, the follow up period will last approximate 11 months till 6 weeks after delivery. If you don't get pregnancy, the outcome of remaining euploid embryo for subjects in the PGS group and the first 3 transfer cycles in the non-PGS group will be followed up. The maximum of time that you will be in the study is 24 months.

What risks/adverse (bad) effects may happen to me by participating in the study?

Below is a table listing all procedures involved in this research and their related discomforts and risks.

Procedures and events	Discomforts and risks
Controlled ovarian hyper-stimulation (COH)	frequent subcutaneous injection, frequent venipuncture, frequent transvaginal ultrasound scan<br>supra-physiologic estrodiol may increase risk of cancer<br>Ovary torsion or ovary disrupture
Ovarian hyperstimulation syndrome (OHSS)	Massive enlargement of your ovaries, fluid in your abdominal cavity, bloating, nausea, vomiting. Severe cases may have fluid in thoracic cavity, breathing difficulties, oliguria even anuria, and may require hospitalization, medication or puncture drainage of fluid in abdomen or thorax. Very severe case may suffer from thrombosis, damage to liver or renal function, even death
Oocyte retrieval	Anesthesia accident, pelvic organ injury, intra-abdominal hemorrhage, puncture site hemorrhage, in serious case surgery or transfusion may be needed, infection
ICSI	Microinjection may injure oocyte, pass unknown disease gene to next generation
Embryo biopsy	Damage to embryo development potential
Embryos transfer	Infection
Embryo frozen and thaw	Embryos development stop, and the survival rate of thawed embryos is 95%
Standard venipuncture for blood work	Slight pain, blue mark at the site of puncture, infection or bleeding at the site
Transvaginal ultrsound	Abdominal or pelvic comfort
Ectopic pregnancy	May require medicine or surgery treatment, in severe case pregnancy site rupture resulting intra-abdominal hemorrhage, even shock or death if treatment delayed
Multiple pregnancy	May require embryo reduction, increase risk of pregnancy complication and fetus abnormalities, Preterm delivery
Infertility treatment	Anxiety or emotional distress at various degree

It is not expected that patients will have all of these complications. You will be assigned to a treatment group by chance.

We will be excluding subjects with a history of major medical morbidity which are contraindication for in-vitro fertilization and pregnancy. Oocyte retrieval, embryo biopsy and transfer will be performed by qualified and experienced physicians. At each investigate site, a responsible investigator or the resident doctor on 24-hour call can be contacted if any adverse event occurs during this study. We will be recording any adverse events, including serious ones. These will

be reviewed every 6 months by the Data Safety Monitor Board, and any serious adverse events will be immediately reviewed.

Every effort to prevent injury as a result of your participation will be taken. It is possible, however, that you could develop complications or injuries as a result of participating in this study. In the adverse event or injury, medical treatment is available but will be provided at the usual charge.

What benefits can I expect?

The treatment you receive may prove to be more effective or to have fewer side effects than the other treatments or other available treatments. The results of this research may guide the future treatment of couples with the same issues.

Can I refuse to be in the study?

Your participation in this study is voluntary. You can refuse to participate. If you decide to take part, you will be given this information sheet to keep and will be asked to sign a consent form. You are free to withdraw (stop your participation) at any time, without giving a reason. There is no penalty, and you will not lose any benefits to which you would otherwise be entitled. If you wish to withdraw from the study, please inform your doctor. The data collected up to the time of your withdrawal will continue to be used.

Your research doctor may take you out of the research study without your permission. Some possible reasons for this are: you experience side effects and continuing the research study would be harmful to your health, or you did not follow the instructions of the study doctor. If your participation in the research ends early, you may be asked to visit the research doctor for a final visit.

During the course of the research you will be provided with any significant new findings that may affect your willingness to continue participating in this research.

Confidentiality and privacy

The investigators always maintain a strict privacy policy. All correspondence to the department is held confidentially; furthermore, at no time will your personal and/or identifying information be shared outside of our organization, for any reason. The records identifying you will be kept confidential and will not be disclosed outside of the study site. If the results of the study are published, your identity will remain confidential.

Subjects have the rights of access to personal data and known study results, if and when needed, you enjoy or may enjoy rights for the protection of the confidentiality of your personal data, such as those regarding the collection, custody, retention, management, control, use (including analysis or comparison).

After the close of this study, the study data and specimens will be retained . The de-identified data and specimens may be shared with other researchers. The specimens may be used for purposes beyond the scope of this study.

A description of this clinical trial will be available on http://www.clinicaltrial.gov. This Web site will not include information that can identify you. At most, the Web site will include a summary of the results. You can search this Web site at any time.

Study Number: __________

Contact Information for Questions or Concerns

You have the right to ask any questions you may have about this research. If you have questions, complaints or concerns or believe you may have developed an injury related to this research, contact investigators at 0531-85651076 or the Ob-Gyn resident doctor on 24-hour call at 0531-85651519.

If you have questions regarding your rights as a research participant or you have concerns or general questions about the research or about your privacy and the use of your personal health

information, contact the research protection advocate in the Reproductive medical hospital affiliated to Shandong University Human Subjects Protection Office at 0531-85651376. You may also call this number if you cannot reach the research team or wish to talk to someone else.

Consent Provision

I have read (or had read to me) the accompanying Subject Information Sheet. I have had time to think about what is involved if I participate in this study. I have had the opportunity to ask questions about the study and have received satisfactory answers. I agree to participate in this study and provide my data from my medical records.

I am aware that:

1. This is a clinical trial study to collect information on the outcome of fresh embryo transfer and frozen-thawed embryo transfer in women requiring IVF/ICSI treatment.

2. My identity and the records identifying me will be kept confidential.

3. My participation in this study is voluntary. I have the right to withdraw my participation any time without penalty or loss of benefits to which I am otherwise entitled.

4. I may at any time ask for additional information from the doctor or a doctor delegated by him/her.

Your signature below means that you have received this information, have asked the questions you currently have about the research and those questions have been answered.

Participant: By signing this consent form, you indicate that you are voluntarily choosing to take part in this research.

I **agree / do not agree*** to save a blood sample for some genetic tests and some further studies.

______________________________ __________
Signature of Participant Date

______________________________ __________
Signature of Participant's husband Date

Person Explaining the Research: Your signature below means that you have explained the research to the participant/participant representative and have answered any questions he/she has about the research.

_________________________________ __________
Signature of person who explained this research Date
(Only approved investigators for this research may explain the research and obtain informed consent.)

Questionnaire

FertiQoL International

Fertility Quality of Life Questionnaire (2008)

For each question, kindly check (tick the box) for the response that most closely reflects how you think and feel. Relate your answers to your current thoughts and feelings. Some questions may relate to your private life, but they are necessary to adequately measure all aspects of your life.

Please complete the items marked with an asterisk (*) only if you have a partner.

	For each question, check the response that is closest to your current thoughts and feelings	Very Poor	Poor	Neither Good nor Poor	Good	Very Good
A	How would you rate your health?	☐	☐	☐	☐	☐
	For each question, check the response that is closest to your current thoughts and feelings	Very Dissatisfied	Dissatisfied	Neither Satisfied Nor Dissatisfied	Satisfied	Very Satisfied
B	Are you satisfied with your quality of life?	☐	☐	☐	☐	☐
	For each question, check the response that is closest to your current thoughts and feelings	Completely	A Great Deal	Moderately	Not Much	Not At All
Q1	Are your attention and concentration impaired by thoughts of infertility?	☐	☐	☐	☐	☐
Q2	Do you think you cannot move ahead with other life goals and plans because of fertility problems?	☐	☐	☐	☐	☐
Q3	Do you feel drained or worn out because of fertility problems?	☐	☐	☐	☐	☐
Q4	Do you feel able to cope with your fertility problems?	☐	☐	☐	☐	☐
	For each question, check the response that is closest to your current thoughts and feelings	Very Dissatisfied	Dissatisfied	Neither Satisfied Nor Dissatisfied	Satisfied	Very Satisfied
Q5	Are you satisfied with the support you receive from friends with regard to your fertility problems?	☐	☐	☐	☐	☐
*Q6	Are you satisfied with your sexual relationship even though you have fertility problems?	☐	☐	☐	☐	☐
	For each question, check the response that is closest to your current thoughts and feelings	Always	Very Often	Quite Often	Seldom	Never
Q7	Do your fertility problems cause feelings of jealousy and resentment?	☐	☐	☐	☐	☐
Q8	Do you experience grief and/or feelings of loss about not being able to have a child (or more children)?	☐	☐	☐	☐	☐
Q9	Do you fluctuate between hope and despair because of fertility problems?	☐	☐	☐	☐	☐
Q10	Are you socially isolated because of fertility problems?	☐	☐	☐	☐	☐
*Q11	Are you and your partner affectionate with each other even though you have fertility problems?	☐	☐	☐	☐	☐
Q12	Do your fertility problems interfere with your day-to-day work or obligations?	☐	☐	☐	☐	☐
Q13	Do you feel uncomfortable attending social situations like holidays and celebrations because of your fertility problems?	☐	☐	☐	☐	☐
Q14	Do you feel your family can understand what you are going through?	☐	☐	☐	☐	☐
	For each question, check the response that is closest to your current thoughts and feelings	An Extreme Amount	Very Much	A Moderate Amount	A Little	Not At All
*Q15	Have fertility problems strengthened your commitment to your partner?	☐	☐	☐	☐	☐
Q16	Do you feel sad and depressed about your fertility problems?	☐	☐	☐	☐	☐
Q17	Do your fertility problems make you inferior to people with children?	☐	☐	☐	☐	☐
Q18	Are you bothered by fatigue because of fertility problems?	☐	☐	☐	☐	☐
*Q19	Have fertility problems had a negative impact on your relationship with your partner?	☐	☐	☐	☐	☐
*Q20	Do you find it difficult to talk to your partner about your feelings related to infertility?	☐	☐	☐	☐	☐
*Q21	Are you content with your relationship even though you have fertility problems?	☐	☐	☐	☐	☐
Q22	Do you feel social pressure on you to have (or have more) children?	☐	☐	☐	☐	☐
Q23	Do your fertility problems make you angry?	☐	☐	☐	☐	☐
Q24	Do you feel pain and physical discomfort because of your fertility problems?	☐	☐	☐	☐	☐

© European Society of Human Reproduction & Embryology and American Society of Reproductive Medicine

Final statistical analysis

Given the possibility of false positive and the decision of not transferring mosaic embryos which are probably to develop into live birth, we hypothesized that the cumulative live birth rate after the transfer of PGT-A-selected euploid blastocysts is not clinically better than that after serially transferring all the 3 untested blastocysts. This study was therefore designed as a non-superiority study. It was estimated the cumulative live birth rate after 3 single embryo transfers was 65% in each group. To be 80% certain that the upper limit of a one-sided 95% confidence interval (CI) would exclude a difference in favor of the PGT-A group by more than 7%, 575 patients were required in each group. In consideration of 5% drop-out rate, a total of 1,208 patients were needed.

Continuous baseline characteristics of PGT-A and IVF groups were represented as means ± SD, and between-group differences were compared by Wilcoxon rank-sum test. Categorical variables were represented as frequencies and percentages, and compared by chi-square test. Fisher's exact test was performed for the number of frequencies less than five. Non-superiority test was used to evaluate the absolute rate differences with a two-sided 90% CI for the primary hypothesis only. Other categorical variables were analyzed by estimation of absolute rate differences and relative rates with a two-sided test. Kaplan-Meier curves were used for the time to live birth. We also conducted secondary analyses based on actual treatment that the patients received, the initial, secondary and third embryo transfer in PGT-A and IVF groups. Subgroup analyses were performed according to different endometrial preparation protocol for frozen embryo transfer and age groups. Two-sided P value of <0.05 were considered significant. All analyses were performed using SAS software (version 9.4, SAS Institute).

Summary of changes

Regarding the cumulative live birth rate, we used non-superiority test and evaluated the absolute rate differences with a two-sided 90% CI, while it was 95% CI in the original analysis plan.
