# Supplementary material for: Excessive Exogenous Gonadotropins and Genetic and Pregnancy Outcomes After Euploidy Embryo Transfer: A Secondary Analysis of a Randomized Clinical Trial
Source: JAMA Netw Open. 2024 Apr 2;7(4):e244438. doi: 10.1001/jamanetworkopen.2024.4438 (PMC10988349; doi:10.1001/jamanetworkopen.2024.4438)
Supplement: Supplement 2. — eTable 1. Endometrial Preparation Protocols for Frozen Embryo Transfers eTable 2. Interaction Between Total Dosage and Duration of Gonadotropins and the Effect on Embryo Mosaicism eTable 3. Binary Logistic Regression of the Effect of Gonadotropin Dosage and Treatment Duration on Cumulative Live Birth Rate eTable 4. Maternal and Neonatal Complications [file jamanetwopen-e244438-s002.pdf]

Supplemental Online Content

Ni T, Zhou W, Liu Y, et al. Excessive exogenous gonadotropins and genetic and pregnancy outcomes after euploidy embryo transfer: a secondary analysis of a randomized clinical trial. *JAMA Netw Open*. 2024;7(4):e244438. doi:10.1001/jamanetworkopen.2024.4438

eTable 1. Endometrial Preparation Protocols for Frozen Embryo Transfers

eTable 2. Interaction Between Total Dosage and Duration of Gonadotropins and the Effect on Embryo Mosaicism

eTable 3. Binary Logistic Regression of the Effect of Gonadotropin Dosage and Treatment Duration on Cumulative Live Birth Rate

eTable 4. Maternal and Neonatal Complications

This supplemental material has been provided by the authors to give readers additional information about their work.

© 2024 Ni T et al. *JAMA Network Open.*

eTable1. The endometrial preparation protocols for frozen embryo transfers.

	≤1500IU, <10d n. (%)	≤1500IU, ≥10d n. (%)	>1500IU, <10d n. (%)	>1500IU, ≥10d n. (%)	p1*	p2*	p3*
natural cycle	78(32.4)	52(39.1)	15(20.3)	83(33.3)			
artificial regimen	149(61.8)	75(56.4)	57(77.0)	143(57.4)	0.40	0.05	0.31
ovulation induction cycle	14(5.8)	6(4.5)	2(2.7)	23(9.2)			

*: All comparisons were calculated with the group1(gonadotropins dosage ≤1500 IU and stimulation duration <10 days) as the reference.

© 2024 Ni T et al. *JAMA Network Open.*

eTable2. The interaction between total dosage and duration of gonadotropins on embryo mosaicism.

	≤1500IU	>1500IU	OR (95%CI)	*p* value for interaction
<10d	56(8.8)	27(14.5)	1.848(1.121,3.044)	0.04
≥10d	44(13.0)	82(12.6)	0.956(0.643,1.423)	

© 2024 Ni T et al. *JAMA Network Open.*

eTable3. The association between gonadotropins dosage, treatment duration, and cumulative live birth rate.

	Live birth n. (%)	Crude OR (95%CI)	Adjusted OR (95%CI)
≤1500IU, <10d	180/211(85.3)	Ref	Ref
≤1500IU, ≥10d	83/113(73.5)	0.476(0.271,0.839)	0.490(0.273,0.882)
>1500IU, <10d	42/62(67.7)	0.362(0.188,0.696)	0.409(0.205,0.818)
>1500IU, ≥10d	161/217(74.2)	0.495(0.304,0.806)	0.526(0.312,0.886)

The analysis was conducted using the binary logistic regression model. Adjusted: Age, BMI, AMH, Endometrial thickness on trigger day.

© 2024 Ni T et al. *JAMA Network Open.*

eTable4. Maternal and neonatal complications

	≤1500IU,<10d (n=211)	≤1500IU,≥10d (n=113)	>1500IU,<10d (n=62)	>1500IU,≥10d （n=217）	Crude OR1(95% CI)	Crude OR2(95% CI)	Crude OR3 (95% CI)
Maternal							
Mild OHSS[a]	17/211(8.1)	11/113(9.7)	1/62(1.6)	17/217(7.8)	1.23(0.56,2.73)	0.19(0.02,1.44)	0.97(0.48,1.96)
Moderate or severe OHSS[a]	6/211(2.8)	0	3/62(4.8)	4/217(1.8)	NA	1.74(0.42,7.16)	0.64(0.18,2.31)
Gestational diabetes mellitus[b]	15/189(7.9)	4/92 (4.3)	7/44(15.9)	21/178(11.8)	0.53(0.17,1.64)	2.20(0.84,5.76)	1.55(0.77,3.12)
Preeclampsia or eclampsia[b]	7/189 (3.7)	0	4/44(9.1)	5/178 (2.8)	NA	2.60(0.73,9.31)	0.75(0.23,2.41)
Gestational hypertension[b]	3/189 (1.6)	1/92 (1.1)	0	6/178 (3.4)	0.68(0.07,6.64)	NA	2.16(0.53,8.78)
Premature rupture of membranes[b]	14/189 (7.4)	10/92 (10.9)	1/44 (2.3)	11/178 (6.2)	1.52(0.65,3.58)	0.29(0.04,2.27)	0.82(0.36,1.87)
Preterm delivery[b]	10/189 (5.3)	7/92 (7.6)	4/44 (9.1)	9/178 (5.1)	1.47(0.54,4.01)	1.79(0.53,6.00)	0.95(0.38,2.40)
Anemia[b]	11/189 (5.8)	4/92 (4.3)	1/44 (2.3)	6/178 (3.4)	0.74(0.23,2.38)	0.38(0.05,2.99)	0.56(0.20,1.56)
Neonatal							
Neonatal hospitalization >3 days[c]	19/180 (10.6)	15/83(18.1)	5/42(11.9)	17/161(10.6)	1.87(0.90,3.89)	1.15(0.40,3.27)	1.00(0.50,2.00)
Neonatal jaundice[c]	37/180 (20.6)	30/83 (36.1)	11/42 (26.2)	37/161 (23.0)	2.19(1.23,3.89)	1.37(0.63,2.98)	1.15(0.69,1.93)
Neonatal infection[c]	6/180 (3.3)	2/83 (2.4)	3/42 (7.1)	5/161 (3.1)	0.72(0.14,3.63)	2.23 (0.54,9.31)	0.93(0.28,3.11)
Low birth weight[c]	4/180 (2.2)	8/83 (9.6)	3/42 (7.1)	5/161 (3.1)	4.69(1.37,16.06)	3.39(0.73,15.73)	1.41(0.37,5.35)
Macrosomia[c]	11/180 (6.1)	7/83 (8.4)	3/42 (7.1)	12/161 (7.5)	1.42(0.53,3.79)	1.18(0.32,4.34)	1.24(0.53,2.89)
Birth weight lower than 5th percentile in singleton[c]	6/180 (3.3)	5/83 (6.0)	0	7/161 (4.3)	1.86(0.55,6.28)	NA	1.32 (0.43,4.01)
Birth weight higher than the 90th percentile in singleton[c]	36/180 (20.0)	14/83 (16.9)	8/42(19.0)	24/161 (14.9)	0.81(0.41,1.60)	0.87(0.37,2.04)	0.68(0.38,1.20)

[a]: Evaluation was performed in total number of patients in this subgroup; [b]: Evaluation was performed in all clinical pregnancies; [c]: Evaluation was performed in all deliveries.
Abbreviations: OHSS, ovarian hyperstimulation syndrome; OR, odds ratios. All comparisons were calculated with the group1(gonadotropins dosage ≤1500 IU and stimulation duration <10 days) as the reference.

© 2024 Ni T et al. *JAMA Network Open.*
