# Supplementary material for: Excessive Exogenous Gonadotropins and Genetic and Pregnancy Outcomes After Euploidy Embryo Transfer: A Secondary Analysis of a Randomized Clinical Trial
Source: JAMA Netw Open. 2024 Apr 2;7(4):e244438. doi: 10.1001/jamanetworkopen.2024.4438 (PMC10988349; doi:10.1001/jamanetworkopen.2024.4438)
Supplement: Supplement 3. — Data Sharing Statement [file jamanetwopen-e244438-s003.pdf]

Data Sharing Statement

Ni. Excessive Exogenous Gonadotropins and Genetic and Pregnancy Outcomes After Euploidy Embryo Transfer: A Secondary Analysis of a Randomized Clinical Trial. *JAMA Netw Open*. Published online April 2, 2024. doi:10.1001/jamanetworkopen.2024.4438

Data
Data available: No

Additional Information
Explanation for why data not available: This is a post hoc analysis of a clinical trial, we have no rights to share the original data.
